# Whole genome sequencing delineates regulatory, copy number, and cryptic splice variants in early onset cardiomyopathy

**DOI:** 10.1038/s41525-022-00288-y

**Published:** 2022-03-14

**Authors:** Robert Lesurf, Abdelrahman Said, Oyediran Akinrinade, Jeroen Breckpot, Kathleen Delfosse, Ting Liu, Roderick Yao, Gabrielle Persad, Fintan McKenna, Ramil R. Noche, Winona Oliveros, Kaia Mattioli, Shreya Shah, Anastasia Miron, Qian Yang, Guoliang Meng, Michelle Chan Seng Yue, Wilson W. L. Sung, Bhooma Thiruvahindrapuram, Jane Lougheed, Erwin Oechslin, Tapas Mondal, Lynn Bergin, John Smythe, Shashank Jayappa, Vinay J. Rao, Jayaprakash Shenthar, Perundurai S. Dhandapany, Christopher Semsarian, Robert G. Weintraub, Richard D. Bagnall, Jodie Ingles, Genomics England Research Consortium, J. C. Ambrose, P. Arumugam, E. L. Baple, M. Bleda, F. Boardman-Pretty, J. M. Boissiere, C. R. Boustred, H. Brittain, M. J. Caulfield, G. C. Chan, C. E. H. Craig, L. C. Daugherty, A. de Burca, A. Devereau, G. Elgar, R. E. Foulger, T. Fowler, P. Furió-Tarí, A. Giess, J. M. Hackett, D. Halai, A. Hamblin, S. Henderson, J. E. Holman, T. J. P. Hubbard, K. Ibáñez, R. Jackson, L. J. Jones, D. Kasperaviciute, M. Kayikci, A. Kousathanas, L. Lahnstein, K. Lawson, S. E. A. Leigh, I. U. S. Leong, F. J. Lopez, F. Maleady-Crowe, J. Mason, E. M. McDonagh, L. Moutsianas, M. Mueller, N. Murugaesu, A. C. Need, C. A. Odhams, A. Orioli, C. Patch, D. Perez-Gil, M. B. Pereira, D. Polychronopoulos, J. Pullinger, T. Rahim, A. Rendon, P. Riesgo-Ferreiro, T. Rogers, M. Ryten, K. Savage, K. Sawant, R. H. Scott, A. Siddiq, A. Sieghart, D. Smedley, K. R. Smith, S. C. Smith, A. Sosinsky, W. Spooner, H. E. Stevens, A. Stuckey, R. Sultana, M. Tanguy, E. R. A. Thomas, S. R. Thompson, C. Tregidgo, A. Tucci, E. Walsh, S. A. Watters, M. J. Welland, E. Williams, K. Witkowska, S. M. Wood, M. Zarowiecki, Marta Melé, Philipp G. Maass, James Ellis, Stephen W. Scherer, Seema Mital

**Affiliations:** 1 Genetics and Genome Biology Program, The Hospital for Sick Children, Toronto, ON Canada; 2 St. George’s University School of Medicine, Grenada, Grenada; 3 Department of Human Genetics, UZ Leuven, Leuven Belgium; 4 Department of Molecular Genetics, University of Toronto, Toronto, ON Canada; 5 Zebrafish Genetics and Disease Models Core, The Hospital for Sick Children, Toronto, ON Canada; 6 Life Sciences Department, Barcelona Supercomputing Center, Barcelona, Catalonia Spain; 7 Division of Genetics, Department of Medicine, Brigham & Women’s Hospital and Harvard Medical School, Boston, MA USA; 8 Developmental and Stem Cell Biology Program, The Hospital for Sick Children, Toronto, ON Canada; 9 Princess Margaret Cancer Center, University Health Network, Toronto, ON Canada; 10 The Centre for Applied Genomics, The Hospital for Sick Children, Toronto, ON Canada; 11 Division of Cardiology, Children’s Hospital of Eastern Ontario, Ottawa, ON Canada; 12 Peter Munk Cardiac Centre, Division of Cardiology, Toronto General Hospital, University of Toronto, Toronto, ON Canada; 13 Department of Pediatrics, Hamilton Health Sciences Centre, Hamilton, ON Canada; 14 Division of Cardiology, London Health Sciences Centre, London, ON Canada; 15 Department of Pediatrics, Kingston General Hospital, Kingston, ON Canada; 16 Cardiovascular Biology and Disease Theme, Institute for Stem Cell Science and Regenerative Medicine, Bangalore (inStem), Bangalore, India; 17 Department of Cardiology, Sri Jayadeva Institute of Cardiovascular Sciences and Research, Bengaluru, India; 18 Agnes Ginges Centre for Molecular Cardiology at Centenary Institute, The University of Sydney, Sydney, Australia; 19 Department of Cardiology, Royal Prince Alfred Hospital, Sydney, Australia; 20 Cardiology Department, Royal Children’s Hospital, Melbourne, Australia; 21 Murdoch Children’s Research Institute and Department of Paediatrics, University of Melbourne, Melbourne, Australia; 22 Cardio Genomics Program at Centenary Institute, The University of Sydney, Sydney, Australia; 23 McLaughlin Centre, University of Toronto, Toronto, ON Canada; 24 Ted Rogers Centre for Heart Research, Toronto, ON Canada; 25 Department of Pediatrics, The Hospital for Sick Children, University of Toronto, Toronto, ON Canada; 26 Genomics England, London, UK; 27 William Harvey Research Institute, Queen Mary University of London, London, EC1M 6BQ UK

**Keywords:** Paediatric research, Gene regulation, Genetic testing

## Abstract

Cardiomyopathy (CMP) is a heritable disorder. Over 50% of cases are gene-elusive on clinical gene panel testing. The contribution of variants in non-coding DNA elements that result in cryptic splicing and regulate gene expression has not been explored. We analyzed whole-genome sequencing (WGS) data in a discovery cohort of 209 pediatric CMP patients and 1953 independent replication genomes and exomes. We searched for protein-coding variants, and non-coding variants predicted to affect the function or expression of genes. Thirty-nine percent of cases harbored pathogenic coding variants in known CMP genes, and 5% harbored high-risk loss-of-function (LoF) variants in additional candidate CMP genes. Fifteen percent harbored high-risk regulatory variants in promoters and enhancers of CMP genes (odds ratio 2.25, *p* = 6.70 × 10^−7^ versus controls). Genes involved in α-dystroglycan glycosylation (*FKTN*, *DTNA*) and desmosomal signaling (*DSC2*, *DSG2*) were most highly enriched for regulatory variants (odds ratio 6.7–58.1). Functional effects were confirmed in patient myocardium and reporter assays in human cardiomyocytes, and in zebrafish CRISPR knockouts. We provide strong evidence for the genomic contribution of functionally active variants in new genes and in regulatory elements of known CMP genes to early onset CMP.

## Introduction

Cardiomyopathy (CMP) is a primarily genetic disease with a prevalence of 1:500 to 1:2500 in the general population and an estimated 20 million people worldwide living with the disease^1^. Several thousand new cases are diagnosed annually in North America^2^. A third are inherited, the remainder is sporadic, with most cases being autosomal dominant caused by rare variants in genes that impact muscle structure and function^3^. There are five phenotypes—hypertrophic (HCM), dilated (DCM), restrictive (RCM), left ventricular non-compaction (LVNC), and arrhythmogenic (ACM) cardiomyopathy. There is considerable genetic overlap between different CMP subtypes. Cardiomyopathy has a high penetrance and is the leading cause of heart failure and sudden cardiac death in childhood^4,5^. The genetic basis of early onset CMP has not been comprehensively evaluated.

While sarcomere genes like *MYH7* and *MYBPC3* explain over 50% of HCM, other CMPs are polygenic. Despite the inclusion of over 100 putative CMP disease genes in clinical testing panels, a majority of cases remain gene-elusive^6,7^. Standard panels only capture small sequence-level variants within the coding regions of known CMP genes and miss hard-to-sequence regions, most intronic splicing events, structural variation, and new candidate CMP genes. Further, these panels do not evaluate the non-coding genome that harbors DNA regulatory sequences including core and proximal promoters and enhancers, as well as distal regulatory elements^8^. Variants in these regulatory elements can disrupt the transcriptional activation process through alterations in chromatin structure, non-coding RNA, transcript stability, and DNA sequence alteration of transcription factor binding sites (TFBS).

Whole-genome sequencing (WGS) studies are beginning to identify novel genetic variants in pediatric and familial disorders^9,10^. In autism spectrum disorder, WGS identified putative non-coding regions as hotspots for de novo germline variants^11^, new candidate risk genes^12^, and novel variant types^13^. Recently, WGS identified a higher burden of de novo variants in the enhancers of disease-associated genes in congenital heart disease patients compared with controls^14^. These studies did not validate the variant impact on endogenous gene expression in patient myocardium, and only 5 of the 31 enhancers identified in congenital heart disease were associated with altered transcription levels of the target genes.

Here, we used WGS to characterize all classes of genetic variation in a well-phenotyped discovery cohort of childhood-onset CMP. WGS identified copy number variants (CNVs), cryptic splicing variants, high-risk regulatory variants associated with known CMP genes, and loss-of-function (LoF) variants in additional candidate genes that would not have been detected on clinical genetic testing. The function of the most important variants was confirmed by measuring endogenous gene expression in patient myocardium, human cardiomyocyte (CM)-based reporter assays, and CRISPR gene editing of zebrafish embryos. This precision variant discovery framework for WGS coupled with comprehensive functional genomics provides an important paradigm for WGS application in CMP.

## Results

**Fig. 1:**
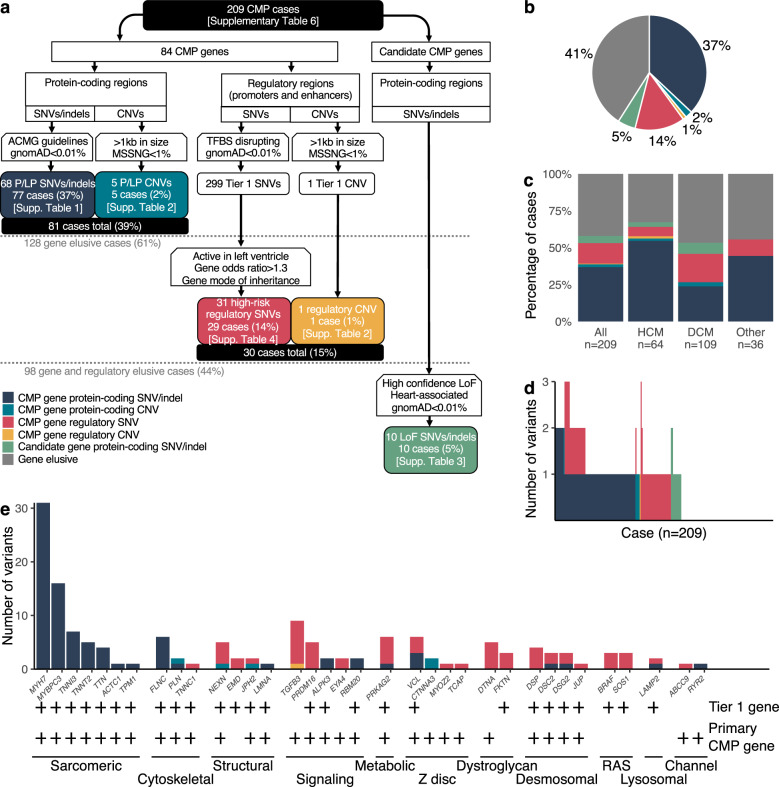
Yield of protein-coding and regulatory variants in 209 unrelated childhood CMP cases. **a** Flow-chart showing the selection process and yield of protein-coding and regulatory variants in the overall cohort and in the gene-elusive subset. Totally, 39% of all cases harbored at least one pathogenic protein-coding variant in a CMP gene; among the remaining 128 gene elusive cases, 15% harbored at least one prioritized high-risk regulatory variant in a CMP gene; and an additional 5% harbored an LoF variant in a new candidate CMP gene. **b** Pie diagram showing the distribution of protein-coding and regulatory variants in CMP genes and LoF variants in new CMP genes across the cohort (*n* = 209). WGS identified putatively pathogenic protein-coding SNVs/indels/CNVs in CMP genes in 39% of cases, high-risk variants in regulatory elements of CMP genes in an additional 15% of cases, and loss of function (LoF) variants in candidate genes in an additional 5% of cases. **c** Variant distribution by CMP subtypes: HCM cases had a higher yield of pathogenic protein-coding variants compared to other CMP subtypes (odds ratio 2.8, CI: 1.5–5.2, *p* = 7.07 × 10^−4^). **d** Variant burden by the patient: 9 cases (4.3%) had multiple protein-coding variants in known CMP genes, 2 cases (1.0%) had multiple prioritized regulatory variants, and 21 cases (10.0%) had both protein-coding and regulatory variants in CMP genes. Regulatory variants in all cases were further prioritized if they were active in human LV, were rare in control subpopulations (Popmax AF < 0.1%), and were associated with genes enriched in cases versus controls with OR ≥ 1.3. **e** Variant distribution by functional gene categories: of all the pathogenic protein-coding variants, 66% was in sarcomere genes which represented a significant enrichment compared to other gene categories (binomial *p* = 3.99 × 10^−29^). Conversely, none of the high-risk regulatory variants were in sarcomere genes. Tier 1 gene and primary CMP gene classifications are denoted by plus symbols. CMP cardiomyopathy, SNV single nucleotide variant, CNV copy number variant, gnomAD Genome Aggregation Database, ACMG American College of Medical Genetics; Association for Molecular Pathology (AMP), TFBS transcription factor binding site, P/LP pathogenic or likely pathogenic, LoF loss of function, HCM hypertrophic cardiomyopathy, DCM dilated cardiomyopathy.

Our overall analysis found that in 209 unrelated probands in the discovery cohort, 77 (37%) cases harbored pathogenic (including likely pathogenic) protein-coding single nucleotide variants (SNVs) and indels, 5 cases (2%) harbored CNVs in known CMP genes, and 10 (5%) cases harbored high-risk LoF variants in additional candidate genes. An additional 15% of cases harbored high-risk variants in regulatory elements of CMP genes. Of these, only 48 variants (31% cases) were known on prior clinical genetic testing. Variant distribution by CMP subtype, by the patient, and by gene category is shown in Fig. 1.

### Protein-coding SNVs, indels, CNVs, and cryptic splice site variants in known CMP genes

The majority (66%) of pathogenic protein-coding variants were in sarcomere genes, a significant enrichment compared to other gene categories (*p* = 3.99 × 10^−29^) (Supplementary Tables 1 and 2). HCM cases had a higher yield of pathogenic variants compared to other phenotypes [odds ratio (OR) = 2.8, 95% confidence intervals (CI): 1.5–5.2, *p* = 7.07 × 10^−4^]. Except for one variant in a secondary CMP gene (*LAMP2*), and three variants in Tier 2 genes (*CTNNA3*×2 and *RYR2*), the remainder were in Tier 1 primary CMP genes. Only three cases harbored homozygous variants. Five cases harbored pathogenic CNVs, none of which were detected on clinical testing. Of note, two pathogenic, heterozygous, intronic cryptic splice variants were identified—*FLNC*:c.7562–2_7581dup and *MYBPC3*:c.1224–52G > A, which was recently reported in South Asian HCM cases^15^. In addition, two pathogenic, heterozygous, protein-coding variants (*MYBPC3*:p.G148R and *VCL*:p.K983fs) were predicted to create new cryptic splice sites, which may represent alternative mechanisms for the functional disruption of these genes. *MYBPC3*:p.G148R was also identified in three HCM cases in the replication cohorts. Overall, WGS detected pathogenic protein-coding variants in an additional 8% of cases not detected on clinical gene panel testing.

**Fig. 2:**
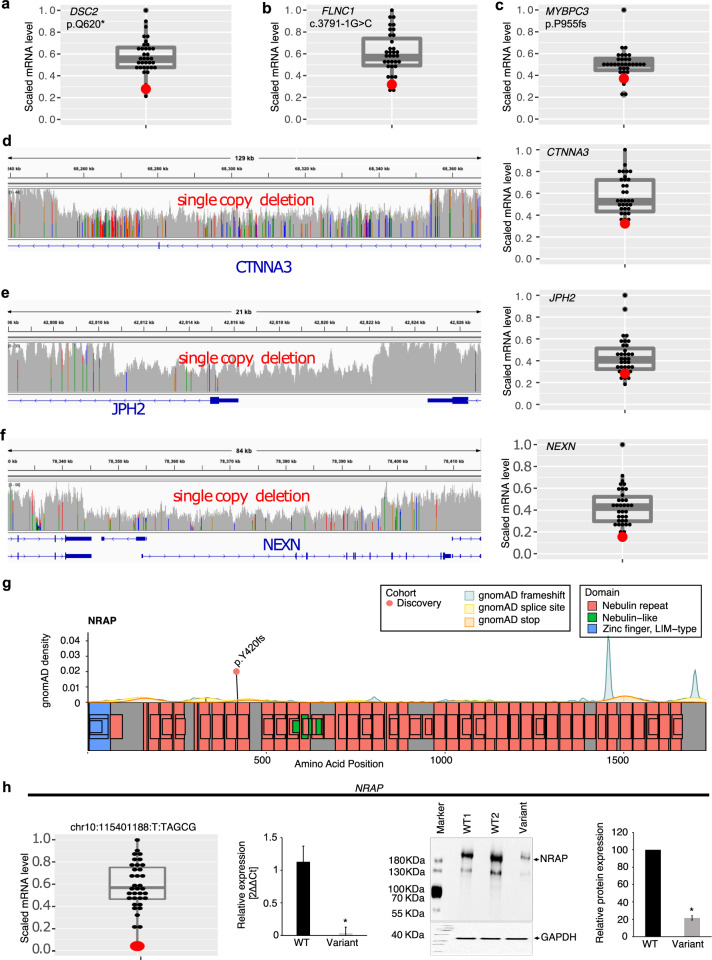
Effect of loss of function and copy number deletions in CMP genes on myocardial gene expression. The figure shows LV myocardial gene expression using RNA sequencing in the patient harboring a loss of function or copy number deletion (red dot) compared to other cases without the variant (gray dots) (*n* = 35 cases). **a**–**c** Three pathogenic loss of function variants predicted to result in nonsense-mediated decay of mRNA. Scaled RPKM expression of target mRNA of variants in *DSC2* (stopgain), *FLNC* (splice acceptor), *MYBPC3* (frameshift deletion) are below the 25th percentile compared to the remaining cohort; **d**–**f** The left panels show the genomic location of three single CNV deletions in *CTNNA3*, *JPH2*, *NEXN* genes. The right panels show scaled RPKM expression of target mRNA below the 25th percentile compared to the remaining cohort. **g** Location of loss of function variant in *NRAP* (ENST00000359988) in the discovery cohort (orange dot). gnomAD background density maps of frameshift, splice site, and premature stop variants are shown. **h** Myocardial *NRAP* expression: RNA-seq analysis demonstrated low *NRAP* mRNA expression (<75th percentile) in the LV myocardium of a DCM patient harboring a homozygous frameshift variant (chr10:115401188_T/TAGCG) (red dot) compared to 34 CMP patients without the variant (black dots). The boxplot shows median expression for the cohort, 25th and 75th percentiles, and lower and upper limit values. qRT-PCR confirmed the reduction of *NRAP* mRNA expression in patients with the variant compared to 2 CMP patients without the variant i.e., WT (*p < 0.05 vs. WT). Western blot confirmed downregulation of *NRAP* protein expression in the patient with the variant compared to three CMP patients without the variant on representative Western blot images (*p < 0.05 vs. WT). RPKM reads per kilobase of transcript, per Million mapped reads, gnomAD Genome Aggregation Database, WT wild-type, mut mutant, 2ΔΔCt the relative fold change in mRNA abundance between samples as a function of polymerase chain reaction thresholds.

A unique feature of our biobank is access to myocardial samples from patients undergoing cardiac surgery or transplantation. LV myocardial mRNA expression was below the 25th percentile in patients harboring LoF SNVs/indels (*DSC2*, *FLNC*, *MYBPC3*) or single deletion CNVs impacting the promoter and first exons of *JPH2* and *NEXN*, and exon 11 of *CTNNA3* compared to controls (Fig. 2d–f). The observed impact of coding variants on endogenous gene expression in the target organ supports the use of patient myocardium to validate variant pathogenicity.

### Protein-coding LoF variants in new candidate CMP genes

WGS provided an opportunity to explore new biologically-relevant genes that are not routinely captured on CMP gene panels. We identified rare LoF variants in 10 candidate genes in CMP patients who were previously gene-elusive (5% of the cohort) (Supplementary Table 3). This included a patient with DCM born of consanguineous parents who was homozygous for an LoF variant in *NRAP* as displayed in Fig. 2g. Homozygous or bi-allelic variants in *NRAP* have been reported as disease-causing in patients with DCM in several studies^16,17^. The patient in our study cohort was diagnosed with severe DCM that progressed to LV RCM physiology requiring cardiac transplantation by 7.7 years of age. Using LV myocardium from this patient, we confirmed that *NRAP* mRNA and protein expression were significantly downregulated compared to controls (Fig. 2h). Several patients in the replication cohorts harbored heterozygous *NRAP* variants but not homozygous or bi-allelic variants.

We identified two patients in our study cohort who were heterozygous for LoF variants in *FHOD3*, one with DCM and one with HCM. Six patients in our replication cohorts also harbored heterozygous LoF *FHOD3* variants. The heterozygous *FHOD3*:p.T502fs variant observed in our patient with DCM was also found in a 100,000 Genomes Project patient with DCM, and three different heterozygous splice variants in *FHOD3* were observed at the same site in patients in the replication cohorts, all with HCM: c.1646 + 1G > C in an Australian patient^18^, c.1646 + 1G > T in a 100,000 Genomes Project patient, and c.1646 + 1G > A in three 100,000 Genomes Project patients, suggesting a variant hotspot (Supplementary Fig. 1a; Supplementary Table 3).

Of other genes harboring LoF variants in our discovery cohort, rare LoF variants in *PDE4DIP* (*n* = 5), *PTGDS* (*n* = 1), *SMURF1* (*n* = 1), and *TRPM4* (*n* = 4) were also seen in our replication cohorts. Variants in these other candidate genes did not show obvious variant hotspots. Details of all LoF variants in these candidate genes in the discovery and replication cohorts are provided in Supplementary Table 3.

For rapid surveillance of gene function in vivo, we induced directed CRISPR–Cas9 knockout of the two most promising candidate genes, *nrap* and *fhod3*, in zebrafish embryos (Supplementary Fig. 1). 22% *nrap* mutants and 26% *fhod3ab* mutant embryos showed an abnormal cardiac phenotype compared to 0% of Cas9 only control, including atrial enlargement and reduced ventricular end-diastolic dimensions suggestive of an RCM CMP phenotype (*p* < 0.01 vs. controls). The embryos were not followed postnatally to determine if the phenotype evolved further. The patient with homozygous *NRAP* LoF variants in our discovery cohort did show a RCM physiology in the context of DCM, while patients with *FHOD3* variants primarily displayed either HCM or DCM consistent with published reports^19–22^. Together with previously published studies^16–23^, these findings provide strong support for a role for LoF variants in *NRAP* and *FHOD3* in causing CMP. Overall, we identified pathogenic or high-risk coding SNVs, indels, and CNVs in known and candidate CMP genes in 44% of cases in our discovery cohort.

### Regulatory variants associated with CMP genes

**Fig. 3:**
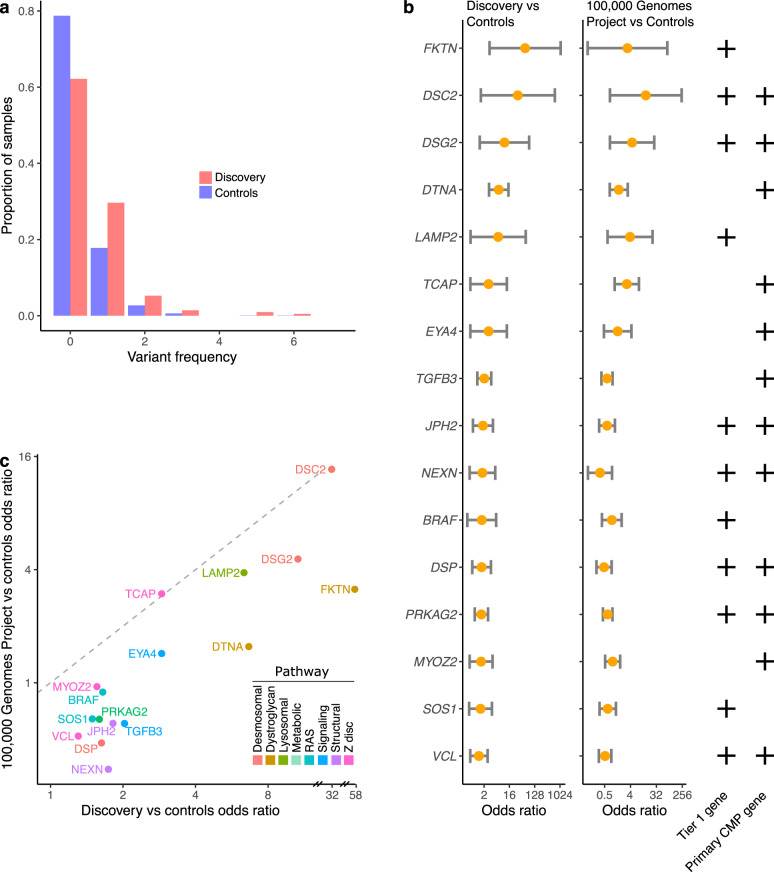
Regulatory variant burden in cases (*n* = 209) and controls (*n* = 1326). **a** There was a significant enrichment of high-risk regulatory variants in CMP genes in the cases (orange) compared to controls (blue) (OR 2.25, 95% CI: 1.65–3.07, *p* = 6.70 × 10^−7^). **b** Burden of regulatory variants genes in cases in the discovery and 100,000 Genomes Project cohorts versus controls. Top 4 genes enriched for regulatory variants compared to controls included *FKTN* (OR = 58.1, CI: 3.1–1083), *DTNA* (OR = 6.7, CI: 3.0–14.8), *DSC2* (OR = 32.0, CI: 1.5–668) and *DSG2* (OR = 10.6, CI: 1.4–81). Tier 1 gene and primary CMP gene classifications are denoted by plus symbols. **c** Replication cohort (*n* = 1266): scatter plot showed a positive correlation between genes enriched for high-risk regulatory variants in the CMP discovery cohort vs the 100,000 Genomes Project replication cohort (Spearman *ρ*^2^ 0.555, *p* = 0.000936) with the top genes being similar in both CMP cohorts (*FKTN*, *DTNA*, *DSC2*, *DSG2*).

Using our previously defined criteria for regulatory variant prioritization, we identified high-risk regulatory variants associated with CMP genes in an additional 15% cases in the overall cohort or 23% of gene-elusive cases. These included 31 prioritized regulatory SNVs in 16 genes (Supplementary Table 4) and a high-risk CNV in a regulatory element of *TGFB3* (Supplementary Table 2). The majority of genes (12 of 16) enriched for high-risk regulatory variants were primary CMP genes. Case–control burden analysis using the ICGC control cohort confirmed an enrichment of regulatory variants in cases compared to controls (OR = 2.25, 95% CI: 1.65–3.07, *p* = 6.70 × 10^−7^) (Fig. 3). The top four enriched genes were in two pathways: (i) α-dystroglycan glycosylation i.e., *FKTN* (OR = 58.1, CI: 3.1–1083) and *DTNA* (OR = 6.7, CI: 3.0–14.8), or (ii) desmosomal i.e., *DSC2* (OR = 32.0, CI: 1.5–668) and *DSG2* (OR = 10.6, CI: 1.4–81). None of the variants were de novo amongst probands with complete trio data. Due to the enrichment of regulatory variants in *FKTN* and *DTNA*, we expanded our search to additional dystroglycanopathy genes (*LARGE1*, *POMT1*, *POMT2*) and identified two regulatory variants of interest in *LARGE1* in gene-elusive cases (Supplementary Table 4). One of the 31 prioritized variants was seen in two unrelated probands (Supplementary Table 4).

In an independent *replication cohort* of 1266 CMP probands from the 100,000 Genomes Project, we found a positive correlation between the discovery and replication cohorts for genes enriched for high-risk regulatory variants (Spearman *ρ*^2^ = 0.555, *p* = 9.36 × 10^−4^) with the top four genes being the same in both cohorts with ORs ranging from 1.6 to 13.7 (Fig. 3c).

Pathogenic protein-coding variants were enriched in genes related to muscle contraction, including binding of actin, troponin C, calmodulin, and protein kinase (Supplementary Fig. 2). In contrast, prioritized regulatory variants were enriched primarily in pathways related to cell adhesion that included genes involved in α-dystroglycan binding and desmosomal signaling. Unlike protein-coding variants, none of the prioritized high-risk regulatory variants were in sarcomere genes. Of note, 32 (15.3%) cases harbored multiple coding and/or regulatory variants in known CMP genes which included 4.3% with multiple protein-coding variants, 1.0% with multiple regulatory variants, and 10.0% with a combination of both variant types (Fig. 1d). Seven genes (*DSC2*, *DSG2*, *JPH2*, *LAMP2*, *NEXN*, *PRKAG2*, *VCL*) harbored high-risk variants in both coding and regulatory regions. Multiple variants were more common in HCM cases compared with other CMP subtypes (OR = 2.67, CI: 1.25–5.70, *p* = 0.013).

### Functional assessment of regulatory variants: association with myocardial gene expression

We selected regulatory variants in seven genes (*BRAF*, *DSP*, *DTNA*, *FKTN*, *LARGE1*, *PRKAG2, TGFB3*) for functional analyses based on the availability of LV myocardium from variant-positive patients. Supplementary Fig. 3 shows high-risk regulatory variants in these eight genes in our discovery cohort and the 100,000 Genomes Project cohort, overlaid on the background of the frequency distribution in the gnomAD reference population^24^. Most regulatory loci were highly constrained in gnomAD. Supplementary Fig. 4 shows the single nucleotide change in the variant of interest in our discovery cohort compared to wild-type sequence and the predicted effect on TF binding motifs.

**Fig. 4:**
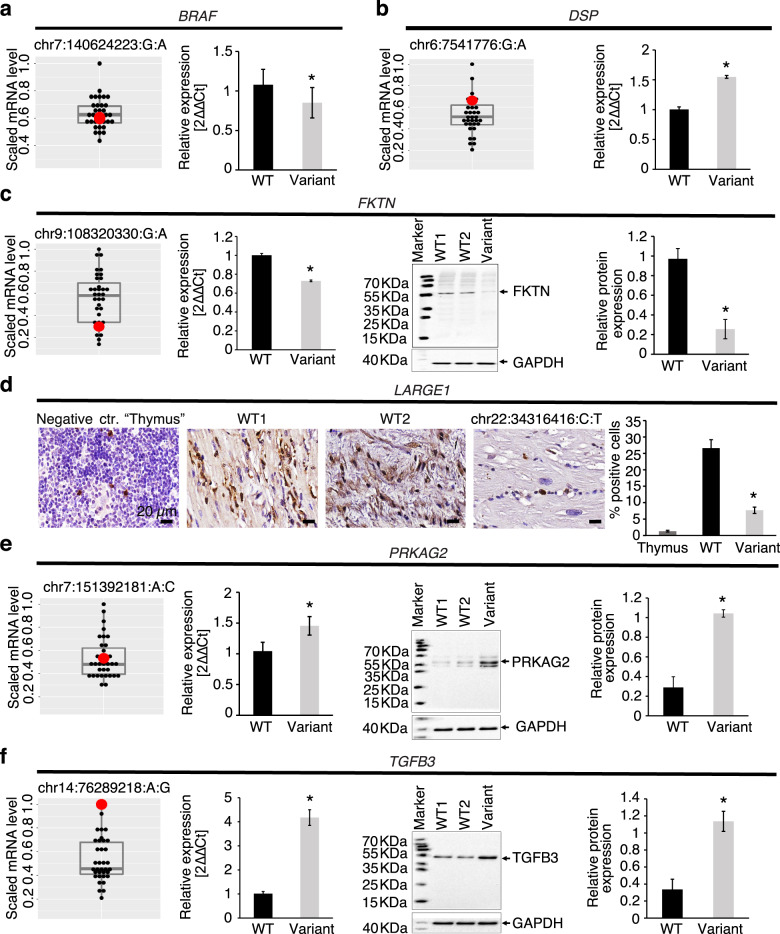
Target gene and protein expression in the LV myocardium of patients harboring regulatory variants. RNA Seq, qRT-PCR, Western blot, and immunohistochemistry were performed in available LV myocardium from CMP patients (*n* = 35) to detect mRNA and protein expression of target genes in patients harboring regulatory variants in *BRAF*, *DSP*, *FKTN*, *LARGE1*, *PRKAG2*, or *TGFB3*. For RNA sequencing data, the target scaled RPKM gene expression was compared between the patient harboring the variant (red dot) and the remainder of the cohort (black dots) using boxplots showing median expression for the cohort, 25th and 75th percentiles, and maximum and minimum values (*n* = 35). For qRT-PCR, Western blot, and immunohistochemistry, target gene or protein expression in the LV myocardium of the patient harboring the variant was compared to wild-type controls including an autopsy sample from an individual without cardiac disease as well as one or more CMP patients that did not harbor any known pathogenic coding or regulatory variants. Three independent experiments were performed per sample with each experiment including three technical replicates per sample. Protein expression level of *GAPDH* as a house keeping gene was used as a loading control for Western blots. Error bars indicate standard deviation between the averages of each independent experiment. **a** *BRAF*: Promoter variant chr7:140624223_G/A was associated with normal *BRAF* mRNA expression on RNAseq, but reduced *BRAF* mRNA expression on qRT-PCR. Promoter variant chr7:140624286_C/T was associated with increased mRNA expression on RNAseq (>75th percentile). **b** *DSP*: Promoter variant (chr6:7541776_G/A) was associated with increased *DSP* mRNA expression on RNAseq (>75th percentile), and on qRT-PCR (*p < 0.05 vs. controls). **c** *FKTN*: Promoter variant 1 (chr9:108320330_G/A) was associated with reduced *FKTN* mRNA expression on RNAseq (<75th percentile), reduced mRNA expression on qRT-PCR (*p* < 0.05 vs. controls), reduced protein expression on Western blot representative images, and reduced relative protein abundance on quantification (*p < 0.05 vs. controls). **d** *LARGE1*: Promoter variant chr22:34316416_C/T was associated with lower perinuclear staining for LARGE1 (brown) (nuclear staining, blue) on representative immunohistochemistry images, and lower % of LARGE1 positive cells in patient myocardium (*p < 0.05 vs. controls). Thymic tissue was used as a negative control. Scale bar = 20 µm. **e** *PRKAG2*: Enhancer variant chr7:151392181_A/C was associated with normal *PRKAG2* mRNA expression on RNAseq, but higher mRNA expression on qRT-PCR (*p < 0.05 vs. controls), higher protein expression on Western blot representative images, and higher relative protein expression on quantification (*p < 0.05 vs. controls). **f** *TGFB3*: Enhancer variant (chr14:76289218_A/G) was associated with higher *TGFB3* mRNA expression on RNAseq, higher mRNA expression on qRT-PCR (*p < 0.05 vs. controls), higher protein expression on Western blot representative images, and higher relative protein abundance on quantification (*p < 0.05 vs. controls). RNA Seq RNA sequencing, WT wild-type, 2ΔΔCt the relative fold-change in mRNA abundance between samples as a function of polymerase chain reaction thresholds.

Myocardial gene expression changes provide critical evidence for the effect of regulatory variants on endogenous gene transcription. When compared to controls, myocardial mRNA and/or protein expression was downregulated in target genes among patients harboring a *BRAF*, *FKTN*, or *LARGE1* promoter variant (Fig. 4). Conversely, target gene expression was upregulated in patients harboring a *DSP* promoter variant, *PRKAG2* enhancer variant, or *TGFB3* enhancer variant. These findings derived directly from the myocardium of patients harboring variants of interest confirmed that SNVs within key regulatory elements had an impact on functional gene products and provide important supporting evidence for a variant effect.

### Functional assessment of regulatory variants: Effect on gene transcription using reporter assays

#### Luciferase reporter assay

**Fig. 5:**
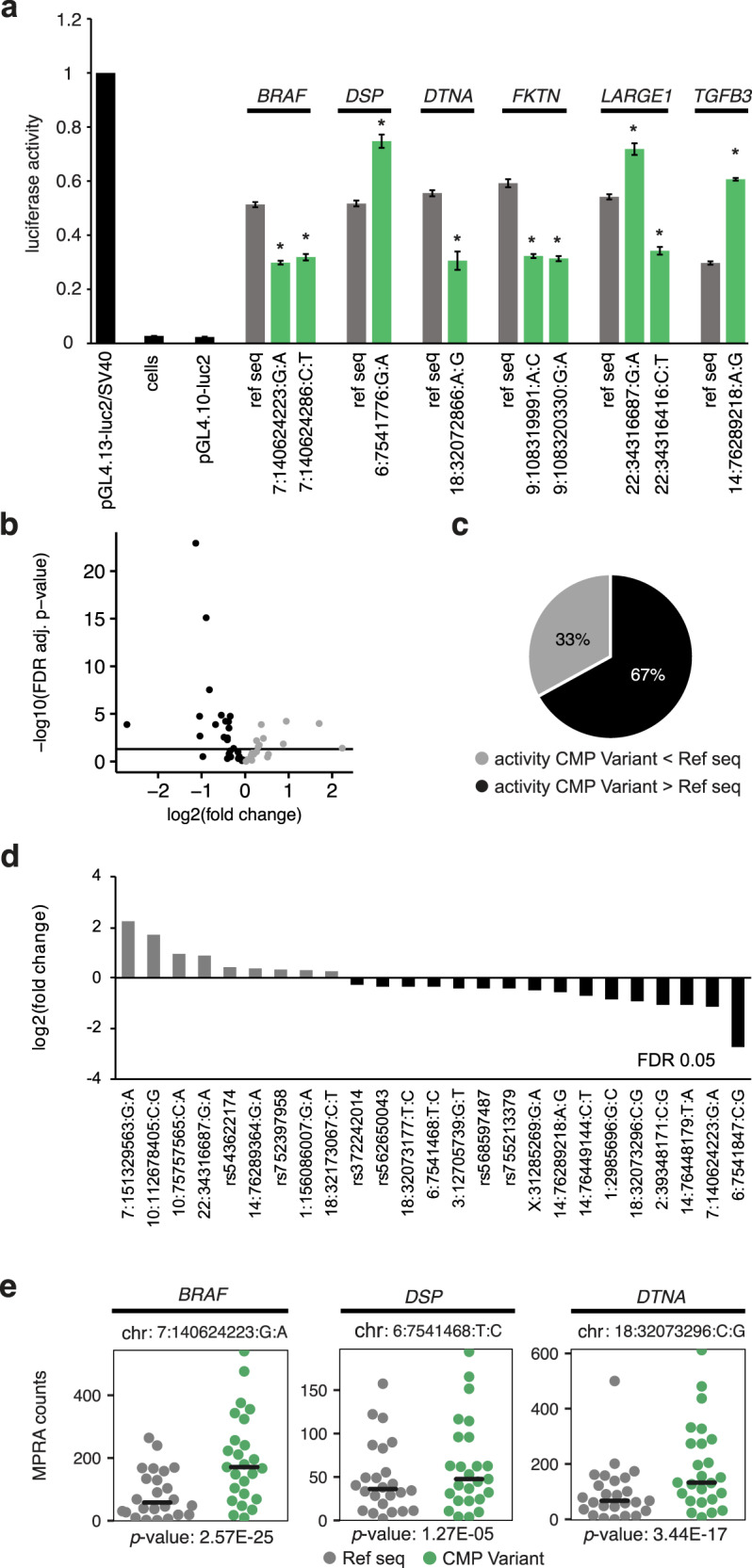
Reporter assays in human iPSC-cardiomyocytes. **a** Luciferase reporter assay showing the effect of regulatory variants on transcription. The cloned promoter variants of *BRAF* (chr7:140624223_G/A), *DTNA* (chr18:32072866_A/G), *FKTN* (chr9:108319991_A/C, chr9:108320330_G/A), and *LARGE1* (chr22:34316416_C/T) reduced luciferase activity compared to reference sequences. The promoter variant of *DSP (*chr6:7541776_G/A*)*, a second promoter variant of *LARGE1* (chr22:34316687_G/A), and an enhancer variant of *TGFB3* (chr14:76289218_A/G) significantly increased luciferase activity compared to reference sequences. *p < 0.05 versus reference sequence. All luciferase reporter assays were performed with three biological replicates, each with three technical replicates. **b** Volcano plot representing the effect of 46 regulatory variants on gene expression using MPRA. Twenty-five variants had significant differences in transcriptional activity between reference and alternative allele (FDR < 0.05, represented by the horizontal black line). Gray = CMP variant activity less than reference allele; black = CMP variant activity more than reference allele. **c** Totally, 67% of significant variants were associated with higher transcription activity of the reference allele. **d** Log2-fold transcriptional activity changes between alternative and reference allele sequences. **e** Representative graphs of MPRA counts of alternative allele (green) versus reference allele sequences (gray) of *BRAF* (chr7:140624223_G/A), *DSP* (chr6:7541468_T/C), and *DTNA* (chr18:32073296_C/G). All MPRA assays were performed in five independent biological replicates. MPRA massively parallel reporter assay, ref seq reference allele sequence, FDR false discovery rate, CMP cardiomyopathy.

Cloned promoter variants of *BRAF*, *DTNA*, *FKTN*, and *LARGE1* reduced luciferase activity compared to reference sequences, while a promoter variant of *DSP*, a second promoter variant of *LARGE1*, and an enhancer variant of *TGFB3* significantly increased luciferase activity in human iPSC-derived CMs (Fig. 5a). This suggests a direct regulatory effect of these SNPs on target gene transcription. Massively parallel reporter assay (MPRA): To assess the functional effect of additional regulatory variants in prioritized genes on transcriptional activity, we used a high throughput MPRA in human iPSC-CMs (Supplementary Fig. 5b–e, Supplementary Table 5)^25^. Of the 46 variants examined, 25 variants (54%) showed significant transcriptional differences between the two alleles (FDR < 0.05) with log2-fold change ranging from −2.72 to +2.23. This represented 23 additional variants with high regulatory activity besides the ones validated in the previous myocardial and luciferase reporter assays. The *BRAF* variant chr7:140624223:G:A had significant but opposite effects on gene expression in the MPRA and luciferase assays, i.e., increased promoter activity on MPRA, but reduced activity on luciferase reporter assay. The MPRA uses short oligonucleotides that provide a high-throughput assay to screen variants for regulatory activity. For quantification and directionality of change in gene expression, luciferase assay findings are considered confirmatory. Overall, of 49 regulatory variants that underwent functional evaluation through a combination of tissue studies, luciferase and/or MPRA reporter assays, 32 (65%) were confirmed to have regulatory activity. Therefore, our findings revealed a significant contribution of regulatory SNVs and CNVs in CMP genes (15% cases), and a small but notable contribution of LoF protein-coding variants in new candidate CMP genes (5% cases) in gene-elusive patients with CMP.

In summary, WGS not only confirmed causal variants previously identified on clinical genetic testing, but detected additional protein-coding variants, cryptic splice site variants and CNVs in CMP genes in another 8% of cases that would not have been found using conventional genetic testing. In the remaining cohort, WGS identified 15% of patients with rare, functionally active high-risk regulatory variants in known CMP genes and 5% patients with high-risk LoF variants in additional candidate CMP genes.

## Discussion

WGS yields a large number of germline protein-coding and regulatory variants. An understanding of their contribution to human disease has been hampered by a lack of systematic bioinformatics and functional approaches tailored to the disease under study. Through WGS, we identified deleterious protein-coding variants in 39% of our CMP cohort, of which 8% were patients who would have been deemed gene-elusive on clinical testing. This increase in diagnostic yield for protein-coding variants with WGS is related to the ability of WGS to detect CNVs, deep intronic cryptic splice site variants, and variants in CMP genes not routinely captured by commercial panels (e.g., *FLNC*)^26^. Additionally, 5% patients harbored deleterious variants in new candidate genes and another 15% harbored high-risk regulatory variants not previously reported in CMP. An important subset of these variants had an effect on exogenous and endogenous gene expression in tissue studies and reporter assays thereby providing strong evidence for their regulatory activity. These validated variants accounted for almost half the number of gene-elusive CMP cases in our cohort.

We applied rigor to our CMP gene selection to avoid including genes with an uncertain association with CMP. We included 84 genes with a reported clinical association with CMP that are offered on clinical gene panels of which 20 genes harbored pathogenic coding variants, the majority of which were primary CMP genes. Only one patient harbored a heterozygous frameshift variant in *LAMP2*, a secondary CMP gene associated with Danon disease. This patient did not have extra-cardiac findings of Danon disease at the time of the last follow-up at age 15 years. While this may reflect incomplete penetrance of extra-cardiac findings, studies have reported that extra-cardiac manifestations can be absent or delayed in those with secondary CMP, including those with Danon disease^27^. This reflects our rationale for including secondary CMP genes and also Tier 2 genes in our study since variants in these genes can contribute to the cardiac phenotype ^28,29^.

An important finding was the identification of CNVs and cryptic splice site variants using WGS. Specifically, we observed both intronic and protein-coding variants predicted to create pathogenic cryptic splice sites in CMP genes. Since clinical laboratories do not routinely test for such variants, it is difficult for them to rely on prior knowledge regarding their pathogenicity. WGS is not only highly sensitive in the detection of such variants but we were also able to confirm changes in myocardial gene expression in patients harboring these variants as further support for their functional effects. These variants represent alternative mechanisms for the functional disruption of genes, and further expand the genetic basis of our CMP cases.

Our study also provided evidence for the contribution of variants in emerging candidate genes that are not yet routinely captured on gene panels^10,16–23^. *NRAP*, *FHOD3*, and *PDE4DIP* are important in the maintenance of the cardiac sarcomere and actin cytoskeleton, and have been associated with CMP in mouse studies and small case series^10,16–23^. LoF variants in these genes were found in DCM and HCM patients in the discovery and replication cohorts, including a patient with a rare homozygous frameshift variant in *NRAP* born of consanguineous parents, consistent with the association of bi-allelic LoF *NRAP* variants in DCM^16^. While most pathogenic *FHOD3* variants reported thus far have been missense variants, we identified several LoF variants that were clustered within the same exon as other previously reported pathogenic variants, including a frameshift variant in our discovery cohort that was also seen in an independent replication cohort. Our findings support a role for LoF variants in *FHOD3* in CMP and are consistent with the relatively low number of *FHOD3* LoF variants observed in controls [gnomAD LoF o/e 0.246 (0.166–0.374)]. Zebrafish models with CRISPR-Cas9 knockouts of *FHOD3* and *NRAP* orthologs further validated that the loss of these genes has a functional effect on the heart. While the zebrafish phenotypes suggested a RCM CMP, the embryos were not followed postnatally to determine if the phenotype evolved to the DCM or HCM phenotype. Also, genetic and phenotypic heterogeneity in CMP is quite common which could also account for variable CMP phenotypes between model organisms and humans, as well as between patients with the same gene defects ^30^.

An exciting finding was the enrichment of high-impact regulatory SNVs and CNVs in cases compared to controls, with 15% cases harboring high-risk regulatory variants. With 65% of all tested regulatory variants validated as being functionally active, it supports our bioinformatics approach to regulatory variant interpretation in CMP. The yield of high-risk regulatory variants was lower in HCM in which protein-coding variants in sarcomere genes already account for a majority of the cases. Sarcomere genes that are more tolerant to haplo-insufficiency were less impacted by regulatory variants. Regulatory variants were enriched mostly in pathways related to α-dystroglycan binding and desmosomal signaling. Although coding variants in these pathways usually cause multi-system involvement, these patients did not manifest systemic features. It is possible that the effect of the observed regulatory variants is restricted to the heart since we only selected variants known to be active in the human LV.

Of the genes enriched for regulatory variants, *DTNA*, *FKTN*, and *LARGE1* are essential for α-dystroglycan function through post-translational glycosylation. Dystroglycan is a central component of the dystrophin-glycoprotein complex, where it plays a role in myocyte, sarcolemma, and sarcomere stability^31^. Disruption of glycosylation has been associated with severe cardiac dysfunction in FKTN or LARGE1-deficient mice and with DCM (with mild to no skeletal muscle involvement)^32–35^. Although *FKTN* is associated with dystroglycanopathy usually in the context of homozygous or compound heterozygous variants^32–34^, the enrichment of heterozygous regulatory variants in our cohort may suggest a contributory role for this gene in CMP. We also found an enrichment of regulatory variants disrupting the expression of desmosomal genes (*DSG2*, *DSC2*, *JUP*, *DSP*) in which both missense and LoF variants have been reported to cause ACM, DCM, and RCM^36^.

A strength of our study was the ability to functionally validate the effect of these regulatory variants on gene and protein expression. We confirmed that the activity of a luciferase reporter gene was altered under the effect of the variant promoter/enhancer sequences compared to wild-type control in human CMs. Moreover, endogenous gene expression was altered in the LV myocardium of patients harboring these variants, a truly unique finding from our study. Together, these findings represent an important advance in our understanding of the genomic architecture of childhood CMP.

Our study has a few limitations. The contribution of regulatory variants may have been underestimated since we did not explore distal enhancers, and because TFBS that do not resemble the consensus sequence could have been misclassified as not being high-risk. As *in silico* predictions improve with time, it will enable more widespread exploration of the regulome for disease variants. The number of probands in whom parents and other family members were available was small which limited our ability to determine variant inheritance and segregation with the disease for all cases. Also, we were not powered to assess the interaction of co-existing regulatory variants on the expressivity of coding variants, and the association of multiple variants with disease severity. Nonetheless, there is growing evidence for the polygenic origins of CMP with recent studies reporting an important contribution of multiple common low impact variants to the penetrance and expressivity of CMP^28,29,37^. However, these studies did not perform a systematic search for rare functional non-coding variants in known genes. In this regard, while regulatory variants identified in our study may or may not be independently causal, their ability to affect the expression of known genes that cause CMP suggests that future efforts should focus on a systematic search for and validation of functionally active regulatory variants that can contribute to the phenotype.

Overall, our findings that high confidence variants identified using in silico prediction models have functional consequences validates our bioinformatics approach to WGS-based variant discovery and makes a strong case for exploring cryptic splice variants, CNVs, variants in new candidate genes, and variants in recurrently altered regulatory elements of CMP genes in order to identify the missing genomic etiology of CMP. In addition to providing a guiding strategy to identify regulatory and new genic variants in childhood CMP, our study provides a framework that can be applied to the search for non-coding variants in other genetic disorders.

## Methods

### Study cohorts for WGS

The *discovery cohort* comprised 209 unrelated probands <21 years old at diagnosis with isolated or primary CMP consented through the Heart Centre Biobank at The Hospital for Sick Children, Toronto^38^. The cases included 52% DCM, 31% HCM, 7% LVNC, 5% RCM, and 2% ACM, with diagnoses based on published clinical criteria (Supplementary Table 6)^39,40^. Patients with secondary CMPs resulting from inborn errors of metabolism, mitochondrial disorders, syndromic, and neuromuscular disorders were excluded. Based on principal components analysis using polymorphic SNVs and data from the 1000 Genomes Project, 67% were of European ancestry, 19% were Asian, 10% were Black. Parents and family members were also recruited whenever possible. This resulted in the availability of 32 probands with complete trios, and 17 with incomplete trios and/or siblings. Collection and use of human DNA and myocardial tissue from CMP cases through the Heart Centre Biobank Registry was approved by the Institutional Research Ethics Boards (Hospital for Sick Children, Children’s Hospital of Eastern Ontario, Toronto General Hospital, London Health Sciences Centre, Kingston General Hospital, and Hamilton Health Sciences Centre) and written informed consent was obtained from all patients and/or their parents/legal guardians^38,41^.

The *control cohort* included 1326 cancer patients with WGS with no known heart disease from the International Cancer Genome Consortium (ICGC)^42^. All genomic data was generated from blood or non-tumor tissue; 747 (56%) were males; 83% were of European ancestry. The diagnoses included 286 pancreatic cancers, 221 brain cancers, 178 prostate cancers, 123 breast cancers, 98 esophageal cancers, 82 liver cancers, 74 renal cancers, 70 skin cancers, 68 ovarian cancers, 64 bone cancers, 37 gastric cancers, 13 oral cancers, and 12 biliary tract cancers.

The *100,000 Genomes Project replication cohort* included 1266 unrelated primary CMP cases with WGS from the 100,000 Genomes Project available through the Genomics England Clinical Interpretation Partnership from version 8 of the main program^43^. LoF (in new candidate genes) and regulatory variant burden analyses were extended to these samples. All cases were required to be probands with WGS data available and have at least one normalized specific disease term matching “cardiomyopathy”. Individuals with additional syndromic Human Phenotype Ontology terms were excluded. The replication cohort included 745 HCM, 355 DCM, 43 LVNC, and 119 ACM subtypes; 22% were less than 21 years old at the time of diagnosis; 62% were male, 82% were of European ancestry.

The *Australian replication cohort* consisted of 528 whole-exome and 59 WGS data derived from 587 unrelated CMP probands recruited at the Genetic Heart Diseases Clinic, Royal Prince Alfred Hospital, Sydney, or the Royal Children’s Hospital, Melbourne^44^. The proband was defined as the first affected family member who sought medical attention at these clinics. Diagnoses of CMP were made based on published clinical criteria^39,40^. Patients provided consent for genetic studies, which were carried out in accordance with the ethics protocol approved by the Sydney Local Health District Ethics Review Committee, Australia, The University of Sydney, Australia, and the Royal Children’s Hospital, Melbourne.

The *South Asian replication cohort* consisted of whole exome sequencing data derived from 100 unrelated HCM probands recruited at the Sri Jayadeva Institute of Cardiovascular Sciences and Research, Bengaluru, India^45^. 65% of cases were male of South Asian ancestry. 58% of cases were childhood-onset (12 ± 4 years) and 42% of cases were adult-onset (29 ± 9 years) cases. All patients provided written informed consent, with appropriate institutional ethics approval.

### Whole-genome sequencing

#### Discovery cohort

WGS was performed on high quality DNA from blood or saliva of probands and their family members to achieve a minimum of 30-fold coverage using Illumina HiSeq X platform through Macrogen, South Korea, and The Centre for Applied Genomics (TCAG, Hospital for Sick Children, Toronto). High-quality paired-end reads (2× 150 bp) were mapped to human genome reference sequence (hg19) using Isaac aligner (https://github.com/Illumina/Isaac4) and short variants were called using Isaac variant caller (https://github.com/sequencing/isaac_variant_caller)^46^. Median sequencing coverage was 31× (range: 20–50×), with WGS quality metrics were calculated using mosdepth (https://github.com/brentp/mosdepth)^47^. Samples with average genome-wide coverage less than 10× were excluded from further analysis. Variants passing default Isaac variant caller quality metrics were annotated using snpEff (v.4.3, https://pcingola.github.io/SnpEff/)^48^ and annovar (v.2016.02.01, https://annovar.openbioinformatics.org/)^49^. Variants used for downstream analysis were further required to have a “PASS” flag in the “FILTER” field. SNVs were additionally required to have a total filtered read depth (“DP”) ≥ 10×, while short insertions and deletions (indels) were additionally required to have a total filtered read depth at the position preceding the indel (“DPI”) ≥ 10×. The total number of SNVs per sample was calculated using bcftools (v1.9, https://samtools.github.io/bcftools/)^50^. Sample genetic ancestry was predicted using somalier (https://github.com/brentp/somalier)^51^. For CNV calling, two read-depth-based algorithms, ERDS estimation by read depth with SNVs (v1.1, https://github.com/igm-team/ERDS)^52^ and CNVnator (v0.3.2, https://github.com/abyzovlab/CNVnator)^53^, were used as previously described^54^. Identified CNV regions were annotated using a custom annotation pipeline developed at TCAG. To increase call confidence, only CNV regions >1 kb in size with at least 50% reciprocal overlap between ERDS and CNVnator calls and <70% overlap with telomeres, centromeres and segmental duplications were included in downstream analyses. To identify de novo variants, we built a full GATK (v4.1.2.0, https://gatk.broadinstitute.org/) best practices^55^ workflow locally for joint calling of short variants (SNVs and indels) within our cohort. Complete parent-offspring trios were available in 32 discovery cohort cases. Paired-end raw reads were first trimmed and cleaned by trimmomatic (v.0.32, http://www.usadellab.org/cms/?page=trimmomatic), then mapped to human reference genome GRCh37 per sample by using bwa (v.0.7.15, https://github.com/lh3/bwa). The reference genome sequence and training dataset were downloaded from the GATK bundle site (ftp.broadinstitute.org/bundle/b37). Mapped reads were realigned and calibrated by base quality score recalibration tools. HaplotypeCaller was used to generate genotype VCF (gVCF) files for each sample. Finally, the gVCF files for all the samples were combined and joint-called by using CombineGVCFs and GenotypeGVCFs tools. In order to filter out probable artifacts in the calls, SNPs and indels were recalibrated separately by variant quality score recalibration (VQSR) tools, and variants that passed VQSR truth sensitivity level 99.5 for SNPs and level 99.0 for indels were retained. To infer possible high confidence de novo sites, we first recalculated phred-scaled genotype likelihoods of the samples by introducing 1000 Genomes project call set (1000G_phase3_v4_20130502) and pedigrees of the trios. These additional data can be used as prior knowledge to recalibrate the confidence of the genotypes, not just calculating a sample’s genotype likelihoods only by its reads. The tool CalculateGenotypePosteriors was applied in this step. Then, we used VariantFiltration to mark out the low Genotype Quality (GQ) sites whose GQ values were lower than 20 and read depths were lower than 10. Lastly, only the sites with all trio numbers ≥ GQ 20 were defined as high confidence de novo variants in the final call set.

#### Control cohort

Data were obtained from the ICGC Data Portal (https://dcc.icgc.org/) Pan-Cancer Analysis of Whole Genomes (PCAWG) section. Samples were aligned to hs37d5 (GRCh37), and germline short variant calls (SNVs) were made using the DKFZ/EMBL variant call pipeline. The “NORMAL” sample calls were extracted and filtered in a comparable way to the discovery cohort: only variants with a “PASS” flag covered by at least 10 reads (DP/DPI ≥ 10) were used for downstream analysis. Variant calls were converted to hg19 using Picard LiftoverVcf (http://broadinstitute.github.io/picard/).

#### 100,000 Genomes Project replication cohort

Where possible, SNVs (indels) were obtained after alignment to the reference genome hg38, otherwise GRCh37 variant calls were used. Variants were filtered to require a “PASS” flag and to have a minimum total read depth (DP/DPI) of 10. hg38 and GRCh37 variant calls were converted to hg19 using Picard LiftoverVcf (http://broadinstitute.github.io/picard/). Variant burden analysis in the cases from the 100,000 Genome Project was performed as previously described by comparing with the ICGC control cohort.

#### Australian replication cohort

Sequencing was performed on an Illumina HiSeq or NextSeq platform as previously described^44^. SNVs and short indels were called using the Genome Analysis Tool Kit (v4.1.1.9, https://gatk.broadinstitute.org/) best practice workflow and annotated using Ensembl Variant Effect Predictor (v97, https://github.com/Ensembl/ensembl-vep). Analysis was restricted to variants in cardiac genes with an allele count <15 in gnomAD v2.1.1 and v3.1 for autosomal dominant genes, or an allele frequency <0.001 for autosomal recessive genes. Variants causing a missense or nonsense change, or that alter the canonical splice dinucleotides, or lead to in-frame or frameshift insertions or deletions, and co-segregate with the disease in affected family members, where available were prioritized. Rare variants of interest were verified using Sanger sequencing.

#### South Asian replication cohort

Samples were sequenced by paired-end, 100-bp reads at service providers including the institutional sequencing facility as previously described^45^. Data were mapped to the human reference genome (GRCh38) using Burrows-Wheeler aligner version 0.7 (BWA-MEM, https://github.com/lh3/bwa). Variant calling was performed using HaplotypeCaller from GATK (v.3.4, https://gatk.broadinstitute.org/). Variants were annotated using web interface of ANNOVAR software (https://annovar.openbioinformatics.org/). Cases were independently analyzed for rare (gnomAD MAF < 0.01%) heterozygous and homozygous loss of function (LoF) variants in candidate genes.

### CMP gene selection strategy

To identify known CMP genes, we curated ten commercially available CMP gene panels to generate a list of putative CMP genes, and retained genes included on at least 2 of 10 panels. Gene panels included Blueprint Genetics Cardiomyopathy Panel, Centogene DCM and HCM Cardiomyopathy Panel, Children’s Hospital of Eastern Ontario Genetics Diagnostic Laboratory Pan Cardiomyopathy Panel, Fulgent Genetics Comprehensive Cardiomyopathy NGS Panel, GeneDx Cardiomyopathy Panel, Invitae Cardiomyopathy Comprehensive Panel, Mayo Clinic Laboratories Comprehensive Cardiomyopathy Multi-Gene Panel, Oregon Health & Science University (OHSU) Knight Diagnostic Laboratories Comprehensive Cardiomyopathy Panel, Partners Personalized Medicine Pan Cardiomyopathy Panel, and PreventionGenetics Pan Cardiomyopathy Panel. Using ClinGen (https://clinicalgenome.org/)^56^, Online Mendelian Inheritance in Man (OMIM, https://www.omim.org/)^57^, ClinVar (https://www.ncbi.nlm.nih.gov/clinvar/)^58^, and manual curation of literature, each gene was classified as either associated with a primary or secondary CMP (i.e., syndromic, metabolic, mitochondrial, or neuromuscular disorders). Genes were further classified based on the strength of the evidence supporting disease association into Tier 1 genes with moderate to definitive evidence, and Tier 2 genes with limited evidence for disease association. Genes with weak, conflicting, or disputed evidence were excluded. Although mitochondrial disorder genes were initially considered, they were ultimately excluded from our final gene set since they are typically associated with autosomal recessive multi-system disease and our cohorts had isolated CMP. The exception was *PRKAG2*, an established Tier 1 gene with known association with HCM. This process yielded a final list of 84 CMP genes (Supplementary Table 7).

### Annotation and classification of protein-coding variants in known CMP genes

Protein-coding rare SNVs, insertion-deletions (indels), and CNVs in CMP genes were classified as pathogenic (including likely pathogenic) using the American College of Medical Genetics (ACMG) and Association for Molecular Pathology (AMP) criteria ^59,60^.

Pathogenicity of missense variants was predicted using prediction scores from at least five prediction tools including SIFT (https://sift.bii.a-star.edu.sg/)^61^, PolyPhen2 (http://genetics.bwh.harvard.edu/pph2/)^62^, MutationTaster2 (http://www.mutationtaster.org/)^63^, Mutation Assessor (http://mutationassessor.org/)^64^, CADD (https://cadd.gs.washington.edu/)^65^, PROVEAN (http://provean.jcvi.org/index.php)^66^, phylogenetic p-value from the PHAST package (http://compgen.cshl.edu/phast/) for multiple alignments of 99 vertebrate genomes to the human genome (phyloP100way_vertebrate)^67^, MetaSVM and MetaLR (https://sites.google.com/site/jpopgen/dbNSFP)^68^. Genomic conservation score was obtained from GERP++ (http://mendel.stanford.edu/SidowLab/downloads/gerp/)^69^, and phastCons (http://compgen.cshl.edu/phast/)^8^. Putative protein-truncating variants predicted to cause LoF including splice-site, nonsense, and frameshift variants were assessed and annotated using LOFTEE tool (https://github.com/konradjk/loftee) as a plugin via Ensembl’s Variant Effect Predictor (VEP) tool (v90, https://github.com/Ensembl/ensembl-vep)^70^. Cryptic splice site variants were identified using SpliceAI software (v1.2.1, https://github.com/Illumina/SpliceAI)^71^ and filtered by a delta score threshold of 0.5 in transcribed regions of genes. ClinVar (https://www.ncbi.nlm.nih.gov/clinvar/)^58^ and Human Gene Mutation Database (HGMD, http://www.hgmd.cf.ac.uk/ac/index.php)^72^ were used to identify previously reported pathogenic or likely pathogenic variants. Rare SNVs and indels were defined by minor allele frequency (MAF) < 0.01% in the Genome Aggregation Database (gnomAD) reference population^24^. Ethnicity-specific MAFs and Popmax 95% confidence interval estimates were compared within gnomAD.

Using human genome CNV map^73^, CNV events overlapping CNV regions that were <30% copy number prone were prioritized for downstream analyses. Rare CNVs were defined as variants occurring at <1% frequency in over 1500 QC pass parental samples from an autism cohort, MSSNG^12^. Rare CNVs >1 kb in size, impacting coding exons were manually inspected using reads from BAM files and were further validated using qPCR with 100% concordance.

Where recommended by ClinGen expert panels, we applied additional ACMG/AMP variant interpretation criteria to genes^74^. For each variant, we assessed if the affected gene was known to be associated with the observed CMP subtype. Each variant’s observed zygosity was compared against the affected gene’s expected disease mode of inheritance. We used complete trios were available to perform *de novo* variant discovery in sporadic cases and to ascertain variant inheritance in familial cases. However, where parents were unavailable or did not consent to study participation, we used singletons for variant identification, but used other available affected and unaffected family members for variant segregation to assist with interpretation of variant pathogenicity. The pathogenicity of variants identified on clinical testing was verified using ClinVar (https://www.ncbi.nlm.nih.gov/clinvar/)^58^ and InterVar (https://github.com/WGLab/InterVar)^75^ classifications where possible. For variants affecting Tier 2 and/or secondary CMP genes, we only retained those considered clinically reportable. Variants in CMP genes that met the pathogenicity criteria described above were considered pathogenic for CMP in genes with strong associations to the disease. These likely pathogenic variants were reviewed and confirmed through independent classification by the institutional molecular genetic screening laboratory and all reportable variants were confirmed using Sanger sequencing where possible.

### Protein-coding LoF variants in new candidate CMP genes

For patients who were gene-elusive, i.e., did not harbor pathogenic or likely pathogenic SNVs, indels, or CNVs, we explored for rare deleterious LoF variants (frameshift, stopgain/stoploss, splicing) in additional candidate genes involved in heart function with moderate-high heart expression, with emerging moderate-strong evidence of association with CMP and/or deemed to be intolerant to haploinsufficiency^24^. To identify such candidate CMP genes that are not usually included in CMP gene panels, we searched for predicted deleterious heterozygous and homozygous LoF variants (i.e., frameshift, nonsense, stopgain, stoploss, and splicing variants) in the in silico exome of CMP cases that did not harbor pathogenic or likely pathogenic SNVs, indels, or CNVs in known CMP genes. LoF variants were identified using LOFTEE (https://github.com/konradjk/loftee)^24,76^. All LoF variants were required to be predicted as high impact by VEP^70^, observed at an allele frequency <0.01% in the gnomAD reference population, observed in <1% of unrelated families in the cohort, and affect genes that are expressed in the human heart. Variants were further prioritized if they were in a highly constrained gene (gnomAD probability of LoF intolerance or pLI > 0.9) and/or were important in heart function. Gene tissue expression level categories were obtained from the Human Protein Atlas (http://www.proteinatlas.org) ^77^.

### Regulatory variants associated with CMP genes

We generated a set of functionally active regulatory elements by mapping non-coding regions in the human heart that putatively regulate the transcription of cardiac-active genes based on experimental evidence and data from the Encyclopedia of DNA Elements project (ENCODE, https://www.encodeproject.org/)^78^, FANTOM project (https://fantom.gsc.riken.jp/)^79^, and Roadmap epigenomics (http://www.roadmapepigenomics.org/)^80^. Discrete regulatory regions that are active in the human heart have been previously identified using these experimental data by Dickel et al^81^. We defined promoter regions of CMP genes by merging the DNase-seq peaks of open chromatin and histone marks specific for promoters and enhancers in these data. Where this information was not available, we defined promoter regions as 1.5 kb upstream and 1 kb downstream of the transcription start site. Enhancers were mapped to genes based on genomic proximity and by using the “False discovery rate-corrected Ordinary least squares with Cross-validation and Shrinkage” database^82^. We focused on promoters and nearby enhancers of known CMP genes rather than the entire genome to avoid false-positive results related to genes with an unclear association with CMP. This provided a total of 2,990,733 base pairs (bp) across 910 unique regulatory regions associated with the 84 CMP genes (Supplementary Table 8). Genes had a median of 8 associated regulatory regions (range 1–38), which encompassed a median of 29,714 bp per gene (range 2236–156,476 bp). The functional impact of rare regulatory variants was assessed based on TFBS creation or disruption scores. The scores for TFBS disruption (motif loss) and TFBS creation (motif gain) were based on combined prediction scores from four different tools—RegulomeDB (https://regulomedb.org/)^83^, motifbreakR (https://bioconductor.org/packages/release/bioc/html/motifbreakR.html)^84^, DeepSEA (http://deepsea.princeton.edu/job/analysis/create/)^85^, and Fathmm-MKL (http://fathmm.biocompute.org.uk/)^86^. We mapped SNVs to these active regulatory regions of CMP genes and defined them as high-risk regulatory variants if they overlapped with established sites in the Ensembl Regulatory Build^87^, were rare (i.e., MAF < 0.01% in gnomAD population controls), and were predicted to alter TF binding by at least 3 of 4 tools that predict if a sequence alteration affects a likely TFBS or has chromatin effects with single-nucleotide sensitivity. The detailed strategy for regulatory variant selection is outlined in Supplementary Fig. 6. We prioritized those variants that were in regulatory elements active in the human left ventricle (LV), were rare in control subpopulations (gnomAD v3.1.2 Popmax AF < 0.1%), were enriched in cases versus controls with OR ≥ 1.3 and were found in gene-elusive cases, i.e., cases that did not harbor pathogenic or likely pathogenic coding variants in known CMP genes. Variants were assessed to determine concordance with expected zygosity and with CMP subtype to be considered contributory to disease. Intergenic and intronic CNVs as well as indels <1 kb overlapping promoter and enhancer regulatory regions were also prioritized. These prioritized high-risk regulatory variants are listed in Supplementary Table 2 and Supplementary Table 4.

### Functional validation of effect on myocardial gene and protein expression

RNA sequencing (RNAseq) was performed in LV myocardial samples available from 35 CMP patients with WGS in our biobank to validate the effect of pathogenic LoF variants, CNVs, and high-risk regulatory variants on target gene expression. LV myocardium was obtained from CMP patients who had consented to biobanking from leftover tissue at the time of cardiac surgery or cardiac transplantation and was immediately snap-frozen in the operating room and stored in liquid nitrogen. RNAseq was performed using Illumina HiSeq 2500 platform at TCAG in 35 LV samples. Total RNA was extracted from LV myocardial samples using the RNeasy Mini kit (QIAGEN, Canada). The generated raw sequence data was filtered according to the procedures described previously^88^. The filtered sequence reads were aligned to the human genome browser UCSC hg19, using Tophat (v.2.0.11, https://ccb.jhu.edu/software/tophat/index.shtml), and processed to extract raw read counts for genes using htseq-count (v.0.6.1p2, https://htseq.readthedocs.io/). Sequencing data were mapped to the human transcriptome using HISAT2 spliced aligner (https://daehwankimlab.github.io/hisat2/)^89^, and the gene expression level was quantified using StringTie (https://ccb.jhu.edu/software/stringtie/)^90^. Reads per kilobase of transcript per million generated were normalized for the size of each library and normalized for the length of the transcripts. Normalized RNAseq data for the genes analyzed in this study are available in Supplementary Table 9. Expression analysis was performed to determine fold-difference in mRNA expression in the variant-positive patient compared to the average values in the remaining cohort (i.e., patients without the candidate SNV or CNV on WGS) ^91^.

For additional confirmation of a difference in the mRNA expression level of the gene harboring the variant compared to the wild type sequences, we determined the relative mRNA expression using qRT-PCR^92^. Total RNA was extracted from patient LV myocardium using mirVana™ PARIS™ RNA and native protein purification Kit (Invitrogen, Carlsbad, California, USA) following the manufacturer’s protocol. The concentration and purity of the RNA were assessed using a Nanodrop 2000c (Thermo Fisher, Waltham, Massachusetts, USA). RNA with an A260/280 ratio of 2.0 ± 0.05 was further evaluated for its integrity using a TapeStation 4200 (Agilent, Santa Clara, California, USA). RNA samples with RNA Integrity number above 5 and rRNA ratio of 1.7–2.0 were used to synthesize complementary DNA (cDNA) using SuperScript IV Reverse Transcriptase (Invitrogen, Carlsbad, California, USA). Specific oligonucleotide primers for each variant (Supplementary Table 10) were designed by primer3-NCBI (https://www.ncbi.nlm.nih.gov/tools/primer-blast/), and synthesized by Integrated DNA technologies (Coralville, Iowa, USA). Glyceraldehyde-3-phosphate dehydrogenase (GAPDH, human) was used as a housekeeping gene for normalization. The qRT-PCR was performed in a ViiA7 qPCR system (Applied Biosystems, Foster City, California, USA) using PowerUp SYBRTM Green Master Mix (Applied Biosystems, Foster City, California, USA). The total volume of the PCR reaction was 10 μl and PCR conditions consisted of a hold stage of 50 °C for 2 min, then 95 °C for 2 min followed by 40 cycles of 15 sec at 95 °C and 15 s at 55–60 °C (Primer Tm dependent) and 72 °C for 1 min. The relative quantification of mRNA was performed using the 2^−ΔΔ*C*T^ method^93^. mRNA expression of target genes in the LV myocardium of the patient harboring the variant was compared to an autopsy sample from an individual without cardiac disease, and from other CMP patients not harboring any known pathogenic coding or regulatory variants. The experiment was performed three independent times and with each experiment, triplicates i.e., three technical replicates, of each sample were prepared and tested.

To determine if change in mRNA expression was associated with a change in protein expression, Western blots were performed to assess myocardial protein expression (Supplementary Table 11)^94,95^. Frozen tissues were homogenized in liquid nitrogen and lysed in radio-immunoprecipitation assay buffer and a protease inhibitor cocktail (Sigma, St. Louis, Missouri, USA). Samples were mixed with loading buffer, heated at 90 °C for 5 min, separated using SDS-blot 4–12% Bis–Tris plus (Invitrogen, Carlsbad, California, USA), and transferred to nitrocellulose membrane. After blocking the membrane with 5% non-fat dry milk in phosphate buffer saline (PBS; pH:7.4), the membrane was incubated with either *FKTN* rabbit monoclonal antibody (ab131280; abcam, Cambridge, UK), rabbit polyclonal *TGFβ3* antibody (ab15537, Abcam, Cambridge, UK), rabbit monoclonal *BRAF* antibody (ab33899, Abcam, Cambridge, UK) or *NRAP* polyclonal antibody (PAS-88772; Invitrogen, Carlsbad, California, USA) in blocking buffer at a dilution 1:1000 for 2 h at room temperature. The reference gene *GAPDH* (ab8245, Abcam, Cambridge, UK) was used as a loading control. After extensive washing of the membrane with PBS/Tween-20, the goat anti-rabbit IgG-HRP and goat anti-mouse IgG-HRP (Invitrogen, Carlsbad, California,) were used as secondary antibodies at a dilution of 1:2000 for 1 h at room temperature. Reactive bands were visualized by ChemiDoc MP imaging system (Bio-Rad, Hercules, California, USA). Protein expression in the LV myocardium of the patient harboring the variant was compared to control samples of other CMP patients who did not harbor this variant. The results were quantified using ImageJ software (http://rsb.info.nih.gov/ij/) and relative protein abundance of the immunoblot signal from each target protein was normalized to the average abundance of the immunoblot signal of control samples. Data were obtained from three independent experiments. All blots or gels derive from the same experiment and were processed in parallel.

Formalin-fixed paraffin-embedded (FFPE) LV tissue from a CMP patient with a *LARGE1* promoter variant and controls without *LARGE1* variants were used for immunohistochemistry (IHC) analysis using standard techniques^96^. FFPE tissue blocks were sectioned at 4 μm, dewaxed in xylene, dehydrated with a serial dilution of ethanol solution and washed with PBS. Antigen retrieval was performed in target retrieval solution (Dako, Burlington, ON, Canada) for 45 min followed by blocking of tissues in 3% hydrogen peroxidase (H_2_O_2_) for 10 minutes. After washing with PBS, tissue sections were incubated for 30 min at room temperature with primary antibody for anti-LARGE1 (PA5-78393, Thermo Fisher, Waltham, Massachusetts, USA) followed by incubation of sections with biotinylated secondary antibody for another 30 min. Immunolabeling was detected using EnVision+ System-HRP DAB kits (Dako, Burlington, ON, Canada). Sections were examined and imaged with a light microscope. Cell nuclei were counterstained with Myer’s Hematoxylin Histological Staining Reagent (Dako, Burlington, ON, Canada). The photographs were analyzed with automated image analysis software (Image J, National Institutes of Health, Bethesda, Maryland). The number of LARGE positive cells was averaged in 10 fields per section and repeated in 3 replicates. Staining was compared between the individual harboring the *LARGE1* variant and the controls.

### Reporter assays in human induced pluripotent stem cells (iPSC)-derived CMs

Gene promoters or enhancer/promoters harboring candidate SNVs and the corresponding control region were cloned into Firefly Luciferase reporters and transfected into human induced pluripotent stem cell (iPSC)-derived CMs to determine the effect of the variants on the transcription activity of the luciferase reporter gene (Supplementary Fig. 5). iPSC derived from peripheral blood lymphocytes of a healthy adult donor (PGP17), were differentiated into CMs using a STEMdiff CM Differentiation Kit^94^. The beating of differentiated iPSC-derived CMs was observed at day 8 post differentiation. Cells were re-seeded at day 16 into 12-well plates for transient transfection. CMs were co-transfected with luciferase constructs at day 20. Transfected cells were harvested 24 h after transfection and firefly and renilla luciferase activity was measured using the Dual-Luciferase® Reporter Assay System.

For functional validation of variant effect on endogenous gene transcription, Dual-Luciferase® Reporter Assay System (Promega, Madison, Wisconsin, USA) was used to test and compare the transcription activity of a luciferase reporter gene under the effect of the variant promoter or promoter/enhancer sequence from the patient, or genome reference sequence of each regulatory region as wild-type control^97,98^. In order to generate the luciferase plasmids harboring the sequence of the regulatory element of the predicted variants and wild-type as a control, the nucleotide sequences of 1.5-kb of the promoter region of *BRAF*, *DSP*, *DTNA*, *FKRP*, *FKTN*, and *LARGE1*, and 2-kb of-enhancer/promoter region of *TGFB3*, containing the strongest transcriptional activation region, were commercially synthesized (Supplementary Table 12) (Synbio Technologies, Monmouth Junction, NJ, USA). The commercial plasmids encoding the respective wild-type, enhancer, or promoter variant sequences were digested with appropriate restriction enzymes and cloned separately into multiple cloning sites of Firefly Luciferase basic vectors (pGL4.10-luc2; Promega, Madison, Wisconsin, USA). Human iPSC-derived CMs were seeded in 12-well plates, and co-transfected with 2 µg firefly luciferase vectors (pGL4.10-luc2; Promega, Madison, Wisconsin, USA) harboring regulatory sequences of wild type, *BRAF*, *DSP*, *DTNA*, *FKRP*, *FKTN*, and *LARGE1* or *TGFB3* variants and 40 ng of Renilla Luciferase control reporter vectors (pRL-TK Vector; Promega, Madison, Wisconsin, USA) for normalization of transfection conditions. At 48 h post-transfection, luminescence was detected with Dual-Luciferase® Reporter (DLR™) assay system. The experiment was performed in three independent replicates and each sample was also tested in triplicate in each experiment. Firefly luciferase was measured, and followed by Renilla luciferase, in the same well. The normalizing activity of the experimental reporter was calculated by dividing the firefly luciferase signal by the internal renilla luciferase signal. Promoter-driven control firefly luciferase vector (pGL4.13-luc2/SV40; Promega, Madison, Wisconsin, USA) was used as a reference.

For massively parallel reporter assays (MPRA), oligonucleotides of 135 bp with 11-bp barcodes were designed and synthesized by TwistBioscience (USA). Variants were centered within the 135 bp oligo. The full list of variants tested can be found in Supplementary Table 5. To control for technical variation and to assess biological relevance, each tested allele was represented a minimum of 25 times, each with a unique barcode. The oligonucleotide library contained 2700 oligos for our genomic variants, 100 oligonucleotides for positive controls, and 1500 oligonucleotides for negative controls i.e., scrambled sequences. These oligonucleotides were part of an oligonucleotide library that included an additional 234,500 sequences as part of a larger study. The cloning strategy of the oligonucleotide library and selection of positive negative controls (300 random sequences, each with 5 barcodes) was performed according to Mattioli et. al^25^. The oligonucleotide library was transfected into five biological replicates of PGP17 iPSC-derived CMs with over 80% transfection efficiency across all replicates, using Lipofectamine Stem Transfection Reagent (STEM00015 Thermo Fisher, Waltham, Massachusetts, USA) (Supplementary Fig. 5b). Forty-eight hours post transfection, total RNA was harvested and DNA contamination was removed using DNase I (18047019, Thermo Fisher, Waltham, Massachusetts, USA). RNA samples with RNA Integrity number >7 were used to synthesize cDNA using SuperScript IV Reverse Transcriptase (Invitrogen, Carlsbad, California, USA). cDNA was used for library synthesis if it lacked plasmid contamination as determined by qRT-PCR performed on a ViiA7 qPCR system (Applied Biosystems, Foster City, California, USA) using PowerUp SYBR^TM^ Green Master Mix (Applied Biosystems, Foster City, California, USA) (Supplementary Fig. 5c). Tag-seq libraries were prepared as previously described^25^, and sequenced with single-end 50 bp reads on the HiSeq2500 platform (TCAG, Hospital for Sick Children, Toronto).

### CRISPR–Cas9 editing to evaluate new candidate CMP gene function in zebrafish embryos

All zebrafish embryo studies were performed at the SickKids Genetics and Disease Models Core (Zebrafish Core), Toronto, and approved by the SickKids Animal Care Committee (Protocol #401951).

All zebrafish guide RNA (gRNA) sequences were adapted from^99^, and are described in Supplementary Table 13. The primer sequences (Supplementary Table 10) were synthesized by Integrated DNA Technologies (IDT, Coralville, Iowa, USA) and used for sgRNA in vitro synthesis, according to the earlier described protocol^99^. Microinjections were performed as described previously^99^ with minor modifications. Briefly, for *nrap* gRNA1, 250 pg of each gRNA with 800 pg Cas9 protein (Alt-R® S.p. Cas9 Nuclease V3, cat #1081058, IDT, Coralville, Iowa, USA) were co-injected into wild-type embryos at the one-cell stage. For the co-injection of 8 gRNAs of *fhod3a* + *b*, gRNA1-gRNA4, 125 pg of each gRNA was injected while the amount of Cas9 protein remained unchanged. The injected embryos were kept in 0.003% Phenylthiourea (PTU) solution and incubated in a dark incubator at 28.5 °C for 3 days. All phenotypic analysis, imaging, DNA extraction, and sequencing were performed at 3-days post fertilization (dpf).

Crude DNA was extracted from whole zebrafish larvae using 1×-PCR buffer (10 mM KCl, 10 mM Tris, PH 8.0; 1.5 mM MgCl2) containing 1 mg/ml proteinase K (Thermo Scientific, Waltham, Massachusetts, USA). The mixture was incubated at 55 °C for 50 min and then 98 °C for 10 min to deactivate proteinase K. To sequence each gRNA region, PCR was performed using Taq DNA polymerase (Bio basic, Markham, ON, Canada). The 25 μl reaction mixture contained 1×-PCR reaction buffer, 2 mM MgCl2, 0.2 mM dNTP, 0.2 mM of each forward and reverse primers, 0.75 U of Taq polymerase, and 1.5 μl of crude DNA (~200 ng). The primer pairs and their corresponding annealing temperatures are summarized in Supplementary Table 10. The PCR reactions were set up as follows: 95 °C for 5 min, followed by 35 cycles of 95 °C for 20 s, annealing temperature for 1 min, 72 °C for 1 min and the final elongation is 72 °C for 5 min. The PCR product was purified using ExoSAP-IT (Applied Biosystems, Foster City, California, USA) following the manufacturer’s instructions and 100 ng of each PCR product was sent for sequencing to TCAG (Toronto, ON, Canada). The sequencing results were analyzed using ICE Analysis (https://ice.synthego.com/#/) or Geneious 9.1.4.

At 3 dpf, pooled RNA samples were collected either from zebrafish larvae injected with gRNAs of target genes or Cas9 only as a control using TRIzol™ Reagent (Invitrogen, Carlsbad, California, USA). First-strand cDNA was synthesized using high capacity cDNA reverse transcription kit (Applied Biosystems, Foster City, California, USA) following the manufacturer’s instructions. These primers were used to amplify two reference genes of *β-actin* and *GAPDH* to normalize data. qRT-PCR assay was performed in a Roche LightCycler 96 machine using PowerUp SYBR Green Master Mix (Applied Biosystems, Foster City, California, USA). The relative expression level was calculated based on two technical repeats using the 2^−ΔΔCT^ method ^93^.

DNA samples were extracted from whole zebrafish larvae at 3 dpf and submitted for Sanger sequencing to TCAG (Toronto, ON, Canada) to confirm cutting efficiency in the exons targeted by *nrap*, *fhod3a*, and *fhod3b* gRNA compared to Cas9 only as a control.

Cardiac phenotyping of zebrafish embryos was performed at 3 dpf to assess cardiac chamber morphology, size and function. For wild field microscope in vivo imaging, 3 dpf zebrafish larvae were anesthetized with 0.02% tricaine and mounted in 3% methylcellulose in 50 mm glass-bottomed dishes. Video imaging was done with the Zeiss AXIO Zoom V16 Microscope using a PlanNeoFluar Z 1×/0.25 FWD 56 mm objective lens under 112× magnification. The Movie Recorder function under Zen pro program was used and approximately 100 frames were captured for each video. All videos were exported at 17 frames per second for further analysis. Images were captured with a Nikon Eclipse Ti microscope under the Nikon A1 plus confocal imaging system using the NIS-Elements program. Atrial area was measured at end-systole, and ventricular area was measured at end-systole and end-diastole with ventricular ejection fraction defined as (end-diastolic area – ventricular systolic area) / ventricular end-systolic area × 100 using ImageJ (https://imagej.nih.gov/ij/).

### Statistical analyses

Figure 1a and Supplementary Fig. 6 depict the workflow for filtering pathogenic and likely pathogenic protein-coding SNVs, indels, CNVs, LoF variants, and high-risk regulatory variants. WGS variant calls were obtained from 1326 patients without heart disease enrolled in the International Cancer Genome Consortium (ICGC)^42^. To compare variant burden between cases and controls for high-risk regulatory variants of CMP genes, variant calls were required to have an allele frequency ≤0.01% in gnomAD. Variants observed in ≥1.5% of samples in the study cohort were excluded from burden testing to reduce false-positive variant calls. Ethnicity-specific allele frequencies were also assessed in the population. All cohorts tested for burden analysis included a majority of samples with European ancestry (Discovery = 67%, Controls = 83%, 100,000 Genomes Project=82%). For genomic burden testing, a case was considered positive if it harbored at least one pathogenic variant (SNV, indel, and/or CNV), otherwise, it was considered negative. *P*-values were calculated using a two-sided Fisher’s exact test. To reduce bias in these calculations and avoid “zero cells” in the contingency tables, 0.5 was added to each observed frequency (Haldane-Anscombe correction). A FDR was applied across genes after removing tests where no variants were observed in any samples. To identify enrichment for sarcomere and cytoskeletal genes among all prioritized regulatory variants, a two-sided binomial test was used. Each variant was considered a “success” if the variant was associated with a sarcomere gene and was considered a “failure” if the variant was associated with a different gene category. The prior probability of “success” was set at 9/84, i.e., equal to the fraction of sarcomere genes among the total set of known CMP genes. Statistical analyses were done using R statistical software (v3.5.1, https://www.r-project.org/).

Pathway enrichment analysis was performed using g:Profiler with default parameters (https://biit.cs.ut.ee/gprofiler)^100^. Queried databases included Gene Ontology (GO), KEGG, and Reactome^101–103^. The protein-coding gene set was ranked according to the total number of pathogenic SNVs, indels, and CNVs observed in our cohort. The regulatory gene set was ranked according to the total number of prioritized regulatory variants observed among cases. Adjusted p-values were calculated using a Bonferroni correction, and only pathways with an adjusted *p*-value < 0.05 were considered significant.

For functional validation including qRT-PCR, Western blots, and IHC, expression levels were compared between a case harboring a variant versus control samples of other CMP cases that did not harbor this variant. An unpaired two-tailed Student’s *t*-test was used to determine differences between groups, with a *p*-value of < 0.05 considered significant.

An unpaired two-tailed Student’s *t*-test was used to compare luciferase activity of the luciferase reporter gene under the effect of the regulatory variant sequence versus the reference sequence of each regulatory region as wild-type control. A *p*-value of < 0.05 was considered significant.

MPRA data were analyzed using MPRAAnalyze software (https://bioconductor.org/packages/release/bioc/html/MPRAnalyze.html)^25,104^ using random oligonucleotide sequences as null distribution. P-values were calculated using a likelihood ratio test with MPRAAnalyze and an FDR < 0.05 was considered significant.

Zebrafish atrial and ventricular sizes and ventricular ejection fraction were compared using an unpaired two-tailed Student’s *t*-test to measure significant differences between mutants (*nrap* and *fhod3*) and controls (Cas9 and wild-type). A *p*-value of < 0.05 was considered significant.

### Reporting summary

Further information on research design is available in the Nature Research Reporting Summary linked to this article.

## Supplementary information

Supplementary Information

Supplementary Tables

Reporting Summary

## Data Availability

Sequencing data are deposited in the European Genome-Phenome Archive (EGA) under accession EGAS00001004929, and are available for download upon approval by the Data Access Committee. Controlled access to the ICGC control cohort data are available through the ICGC Data Portal upon approval from their Data Access Compliance Office. The 100,000 Genomes Project replication cohort are available to GeCIP researchers and trainees using the Genomics England Research Environment upon institutional approval through their Participation Agreement process. Additional data generated or analyzed during this study are included in the supplementary information files, and additional raw data used for figures and results are available from the corresponding author on reasonable request.

## Code availability

All computational tools used in this study are available for download as commercial or open-source software and are detailed in Methods. Detailed parameters of all functions used for each tool are available in the Methods.

## References

[1] Maron BJ, Rowin EJ, Maron MS (2018). Global burden of hypertrophic cardiomyopathy. JACC Heart Fail..

[2] Semsarian C, Ingles J, Maron MS, Maron BJ (2015). New perspectives on the prevalence of hypertrophic cardiomyopathy. J. Am. Coll. Cardiol..

[3] Jacoby D, McKenna WJ (2012). Genetics of inherited cardiomyopathy. Eur. Heart J..

[4] Miron A (2020). A validated model for sudden cardiac death risk prediction in pediatric hypertrophic cardiomyopathy. Circulation.

[5] Mathew J (2018). Utility of genetics for risk stratification in pediatric hypertrophic cardiomyopathy. Clin. Genet..

[6] Alfares AA (2015). Results of clinical genetic testing of 2,912 probands with hypertrophic cardiomyopathy: expanded panels offer limited additional sensitivity. Genet. Med..

[7] Ouellette AC (2018). Clinical genetic testing in pediatric cardiomyopathy: Is bigger better?. Clin. Genet..

[8] Siepel A (2005). Evolutionarily conserved elements in vertebrate, insect, worm, and yeast genomes. Genome Res..

[9] Lionel AC (2018). Improved diagnostic yield compared with targeted gene sequencing panels suggests a role for whole-genome sequencing as a first-tier genetic test. Genet. Med..

[10] Minoche AE (2019). Genome sequencing as a first-line genetic test in familial dilated cardiomyopathy. Genet. Med..

[11] Yuen RKC (2016). Genome-wide characteristics of de novo mutations in autism. NPJ Genomic Med..

[12] C Yuen RK (2017). Whole genome sequencing resource identifies 18 new candidate genes for autism spectrum disorder. Nat. Neurosci..

[13] Trost B (2020). Genome-wide detection of tandem DNA repeats that are expanded in autism. Nature.

[14] Richter F (2020). Genomic analyses implicate noncoding de novo variants in congenital heart disease. Nat. Genet..

[15] Harper AR (2020). Reevaluation of the South Asian MYBPC3Δ25bp intronic deletion in hypertrophic cardiomyopathy. Circ. Genomic Precis. Med..

[16] Koskenvuo JW (2021). Biallelic loss-of-function in NRAP is a cause of recessive dilated cardiomyopathy. PloS ONE.

[17] Truszkowska GT (2017). Homozygous truncating mutation in NRAP gene identified by whole exome sequencing in a patient with dilated cardiomyopathy. Sci. Rep..

[18] Semsarian C, Ingles J, Bagnall RD (2019). Revisiting genome sequencing data in light of novel disease gene associations. J. Am. Coll. Cardiol..

[19] Wooten EC (2013). Formin homology 2 domain containing 3 variants associated with hypertrophic cardiomyopathy. Circ. Cardiovasc. Genet..

[20] Ochoa JP (2018). Formin homology 2 domain containing 3 (FHOD3) is a genetic basis for hypertrophic cardiomyopathy. J. Am. Coll. Cardiol..

[21] Arimura T (2013). Dilated cardiomyopathy-associated FHOD3 variant impairs the ability to induce activation of transcription factor serum response factor. Circ. J..

[22] Esslinger U (2017). Exome-wide association study reveals novel susceptibility genes to sporadic dilated cardiomyopathy. PloS ONE.

[23] Matsuyama S (2018). Interaction between cardiac myosin-binding protein C and formin Fhod3. Proc. Natl Acad. Sci. USA.

[24] Karczewski KJ (2020). The mutational constraint spectrum quantified from variation in 141,456 humans. Nature.

[25] Mattioli K (2019). High-throughput functional analysis of lncRNA core promoters elucidates rules governing tissue specificity. Genome Res..

[26] Gross AM (2019). Copy-number variants in clinical genome sequencing: deployment and interpretation for rare and undiagnosed disease. Genet. Med..

[27] Lotan D (2020). Clinical profile of cardiac involvement in Danon disease: a multicenter European Registry. Circ. Genomic Precis. Med..

[28] Harper AR (2021). Common genetic variants and modifiable risk factors underpin hypertrophic cardiomyopathy susceptibility and expressivity. Nat. Genet..

[29] Tadros R (2021). Shared genetic pathways contribute to risk of hypertrophic and dilated cardiomyopathies with opposite directions of effect. Nat. Genet..

[30] Walsh R, Offerhaus JA, Tadros R, Bezzina CR (2021). Minor hypertrophic cardiomyopathy genes, major insights into the genetics of cardiomyopathies. Nat. Rev. Cardiol..

[31] Johnson EK (2012). Proteomic analysis reveals new cardiac-specific dystrophin-associated proteins. PloS ONE.

[32] Murakami T (2006). Fukutin gene mutations cause dilated cardiomyopathy with minimal muscle weakness. Ann. Neurol..

[33] Arimura T (2009). Mutational analysis of fukutin gene in dilated cardiomyopathy and hypertrophic cardiomyopathy. Circ. J..

[34] Ujihara Y (2019). Elimination of fukutin reveals cellular and molecular pathomechanisms in muscular dystrophy-associated heart failure. Nat. Commun..

[35] Holzfeind PJ (2002). Skeletal, cardiac and tongue muscle pathology, defective retinal transmission, and neuronal migration defects in the Large(myd) mouse defines a natural model for glycosylation-deficient muscle–eye–brain disorders. Hum. Mol. Genet..

[36] James CA, Syrris P, van Tintelen JP, Calkins H (2020). The role of genetics in cardiovascular disease: arrhythmogenic cardiomyopathy. Eur. Heart J..

[37] Walsh R, Tadros R, Bezzina CR (2020). When genetic burden reaches threshold. Eur. Heart J..

[38] Papaz T (2019). Return of genetic and genomic research findings: experience of a pediatric biorepository. BMC Med. Genomics.

[39] Elliott P (2008). Classification of the cardiomyopathies: a position statement from the European Society Of Cardiology Working Group on Myocardial and Pericardial Diseases. Eur. Heart J..

[40] Maron BJ (2006). Contemporary definitions and classification of the cardiomyopathies: an American Heart Association Scientific Statement from the Council on Clinical Cardiology, Heart Failure and Transplantation Committee; Quality of Care and Outcomes Research and Functional Genomics and Translational Biology Interdisciplinary Working Groups; and Council on Epidemiology and Prevention. Circulation.

[41] Papaz T (2012). Factors influencing participation in a population-based biorepository for childhood heart disease. Pediatrics.

[42] International Cancer Genome Consortium. (2010). International network of cancer genome projects. Nature.

[43] Caulfield M (2019). The National Genomics Research and Healthcare Knowledgebase.

[44] Bagnall RD (2018). Whole Genome Sequencing Improves Outcomes of Genetic Testing in Patients With Hypertrophic Cardiomyopathy. J. Am. Coll. Cardiol..

[45] Dhandapany PS (2021). Adiponectin receptor 1 variants contribute to hypertrophic cardiomyopathy that can be reversed by rapamycin. Sci. Adv.

[46] Raczy C (2013). Isaac: ultra-fast whole-genome secondary analysis on Illumina sequencing platforms. Bioinformatics.

[47] Pedersen BS, Quinlan AR (2018). Mosdepth: quick coverage calculation for genomes and exomes. Bioinformatics.

[48] Cingolani P (2012). A program for annotating and predicting the effects of single nucleotide polymorphisms, SnpEff: SNPs in the genome of Drosophila melanogaster strain w1118; iso-2; iso-3. Fly.

[49] Wang K, Li M, Hakonarson H (2010). ANNOVAR: functional annotation of genetic variants from high-throughput sequencing data. Nucleic Acids Res..

[50] Danecek P (2011). The variant call format and VCFtools. Bioinformatics.

[51] Pedersen BS (2020). Somalier: rapid relatedness estimation for cancer and germline studies using efficient genome sketches. Genome Med..

[52] Zhu M (2012). Using ERDS to infer copy-number variants in high-coverage genomes. Am. J. Hum. Genet..

[53] Abyzov A, Urban AE, Snyder M, Gerstein M (2011). CNVnator: an approach to discover, genotype, and characterize typical and atypical CNVs from family and population genome sequencing. Genome Res..

[54] Trost B (2018). A comprehensive workflow for read depth-based identification of copy-number variation from whole-genome sequence data. Am. J. Hum. Genet..

[55] Poplin, R. et al. Scaling accurate genetic variant discovery to tens of thousands of samples, Preprint at *bioRxiv* 10.1101/201178 (2018).

[56] Rehm HL (2015). ClinGen—the Clinical Genome Resource. N. Engl. J. Med..

[57] Amberger JS, Bocchini CA, Scott AF, Hamosh A (2019). OMIM.org: leveraging knowledge across phenotype-gene relationships. Nucleic Acids Res..

[58] Landrum MJ (2016). ClinVar: public archive of interpretations of clinically relevant variants. Nucleic Acids Res..

[59] Richards S (2015). Standards and guidelines for the interpretation of sequence variants: a joint consensus recommendation of the American College of Medical Genetics and Genomics and the Association for Molecular Pathology. Genet. Med..

[60] Riggs ER (2020). Technical standards for the interpretation and reporting of constitutional copy-number variants: a joint consensus recommendation of the American College of Medical Genetics and Genomics (ACMG) and the Clinical Genome Resource (ClinGen). Genet. Med..

[61] Ng PC, Henikoff S (2003). SIFT: predicting amino acid changes that affect protein function. Nucleic Acids Res..

[62] Adzhubei, I., Jordan, D. M. & Sunyaev, S. R. Predicting functional effect of human missense mutations using PolyPhen-2. *Curr. Protoc. Hum. Genet*. **Chapter 7**, Unit 7.20 (2013). 10.1002/0471142905.hg0720s76 PMC4480630 23315928

[63] Schwarz JM, Cooper DN, Schuelke M, Seelow D (2014). MutationTaster2: mutation prediction for the deep-sequencing age. Nat. Methods.

[64] Reva B, Antipin Y, Sander C (2011). Predicting the functional impact of protein mutations: application to cancer genomics. Nucleic Acids Res..

[65] Kircher M (2014). A general framework for estimating the relative pathogenicity of human genetic variants. Nat. Genet..

[66] Choi Y, Sims GE, Murphy S, Miller JR, Chan AP (2012). Predicting the functional effect of amino acid substitutions and indels. PloS ONE.

[67] Hubisz MJ, Pollard KS, Siepel A (2011). PHAST and RPHAST: phylogenetic analysis with space/time models. Brief Bioinform..

[68] Dong C (2015). Comparison and integration of deleteriousness prediction methods for nonsynonymous SNVs in whole exome sequencing studies. Hum. Mol. Genet..

[69] Davydov EV (2010). Identifying a high fraction of the human genome to be under selective constraint using GERP++. PLoS Comput. Biol..

[70] McLaren W (2010). Deriving the consequences of genomic variants with the Ensembl API and SNP Effect Predictor. Bioinformatics.

[71] Jaganathan K (2019). Predicting splicing from primary sequence with deep learning. Cell.

[72] Stenson PD (2017). The Human Gene Mutation Database: towards a comprehensive repository of inherited mutation data for medical research, genetic diagnosis and next-generation sequencing studies. Hum. Genet..

[73] Zarrei M, MacDonald JR, Merico D, Scherer SW (2015). A copy number variation map of the human genome. Nat. Rev. Genet..

[74] Morales A (2020). Variant Interpretation for Dilated Cardiomyopathy: refinement of the American College of Medical Genetics and Genomics/ClinGen Guidelines for the DCM Precision Medicine Study. Circ. Genomic Precis. Med..

[75] Li Q, Wang K (2017). InterVar: clinical interpretation of genetic variants by the 2015 ACMG-AMP guidelines. Am. J. Hum. Genet..

[76] Cassa CA (2017). Estimating the selective effects of heterozygous protein-truncating variants from human exome data. Nat. Genet..

[77] Uhlén M (2015). Proteomics. Tissue-based map of the human proteome. Science.

[78] ENCODE Project Consortium. (2012). An integrated encyclopedia of DNA elements in the human genome. Nature.

[79] Andersson R (2014). An atlas of active enhancers across human cell types and tissues. Nature.

[80] Roadmap Epigenomics Consortium. (2015). Integrative analysis of 111 reference human epigenomes. Nature.

[81] Dickel DE (2016). Genome-wide compendium and functional assessment of in vivo heart enhancers. Nat. Commun..

[82] Hait TA, Amar D, Shamir R, Elkon R (2018). FOCS: a novel method for analyzing enhancer and gene activity patterns infers an extensive enhancer-promoter map. Genome Biol..

[83] Boyle AP (2012). Annotation of functional variation in personal genomes using RegulomeDB. Genome Res..

[84] Coetzee SG, Coetzee GA, Hazelett DJ (2015). motifbreakR: an R/Bioconductor package for predicting variant effects at transcription factor binding sites. Bioinformatics.

[85] Shihab HA (2015). An integrative approach to predicting the functional effects of non-coding and coding sequence variation. Bioinformatics.

[86] Shihab HA (2014). Ranking non-synonymous single nucleotide polymorphisms based on disease concepts. Hum. Genomics.

[87] Zerbino DR, Wilder SP, Johnson N, Juettemann T, Flicek PR (2015). The ensembl regulatory build. Genome Biol..

[88] Gao J, Collyer J, Wang M, Sun F, Xu F (2020). Genetic dissection of hypertrophic cardiomyopathy with myocardial RNA-seq. Int. J. Mol. Sci..

[89] Kim D, Langmead B, Salzberg SL (2015). HISAT: a fast spliced aligner with low memory requirements. Nat. Methods.

[90] Pertea M (2015). StringTie enables improved reconstruction of a transcriptome from RNA-seq reads. Nat. Biotechnol..

[91] Anders S, Huber W (2010). Differential expression analysis for sequence count data. Genome Biol..

[92] Xie H (2018). Identification of TBX2 and TBX3 variants in patients with conotruncal heart defects by target sequencing. Hum. Genomics.

[93] Livak KJ, Schmittgen TD (2001). Analysis of relative gene expression data using real-time quantitative PCR and the 2(-Delta Delta C(T)) Method. Methods.

[94] Hildebrandt MR (2019). Precision health resource of control iPSC lines for versatile multilineage differentiation. Stem Cell Rep..

[95] Patel P, Kuzmanov U, Mital S (2016). Avoiding false discovery in biomarker research. BMC Biochem..

[96] Visonà SD (2018). Diagnosis of sudden cardiac death due to early myocardial ischemia: an ultrastructural and immunohistochemical study. Eur. J. Histochem.

[97] Madan N (2019). Functionalization of CD36 cardiovascular disease and expression associated variants by interdisciplinary high throughput analysis. PLoS Genet..

[98] Kapoor A (2019). Multiple SCN5A variant enhancers modulate its cardiac gene expression and the QT interval. Proc. Natl Acad. Sci. USA.

[99] Wu RS (2018). A rapid method for directed gene knockout for screening in G0 zebrafish. Dev. Cell.

[100] Raudvere U (2019). g:Profiler: a web server for functional enrichment analysis and conversions of gene lists (2019 update). Nucleic Acids Res..

[101] Ashburner M (2000). Gene ontology: tool for the unification of biology. The Gene Ontology Consortium. Nat. Genet..

[102] The Gene Ontology Consortium. (2019). The Gene Ontology Resource: 20 years and still GOing strong. Nucleic Acids Res..

[103] Jassal B (2020). The reactome pathway knowledgebase. Nucleic Acids Res..

[104] Ashuach T (2019). MPRAnalyze: statistical framework for massively parallel reporter assays. Genome Biol..

