# Supplementary material for: Whole genome sequencing delineates regulatory, copy number, and cryptic splice variants in early onset cardiomyopathy
Source: NPJ Genom Med. 2022 Mar 14;7:18. doi: 10.1038/s41525-022-00288-y (PMC8921194; doi:10.1038/s41525-022-00288-y)
Supplement: Supplementary file 1 — Supplementary Information [file 41525_2022_288_MOESM1_ESM.pdf]

SUPPLEMENTARY INFORMATION

SUPPLEMENTARY FIGURES

Supplementary Figure 1

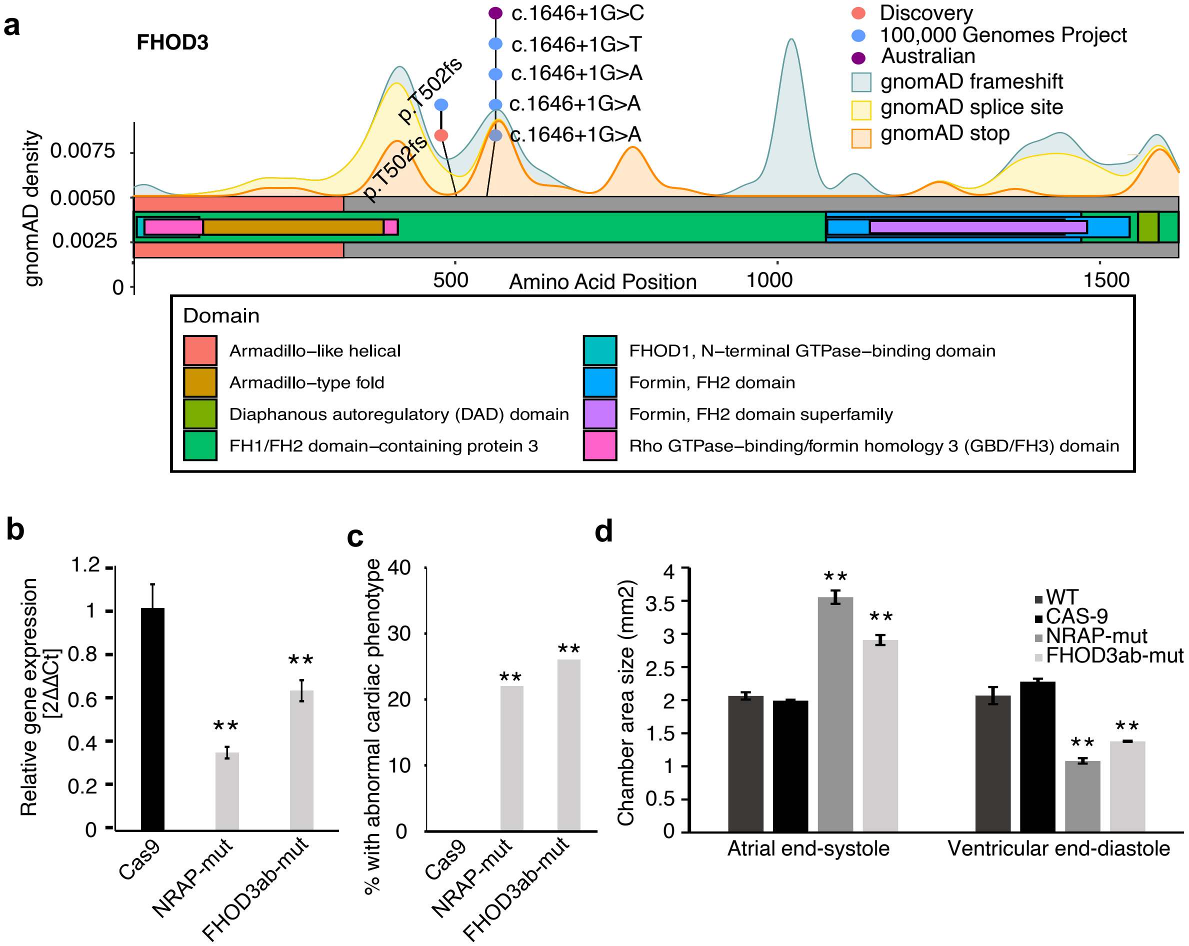

Supplementary Figure 1: Loss of function variants in candidate CMP genes: Location of loss of function variants in (**a**) *FHOD3* (ENST00000590592) in 209 CMP cases in the discovery cohort (orange dot), and in 1326 CMP cases in the 100,000 Genomes Project replication cohort (blue dots). (**b-g**) Zebrafish embryos Zebrafish embryos at 1-cell stage were injected with 4 CRISPR-Cas9 guide RNA complexes to induce knockout of 2 genes, *nrap* and *fhod3ab*. (**b**) qRT-PCR showed a 35-49% downregulation of target mRNA expression in pooled samples of *nrap* and *fhod3ab* mutants compared to WT controls and Cas9 only controls (n=3 independent replicates per gene) (**p<0.01 versus controls). (**c**) 22% *nrap* mutants and 26% *fhod3ab* mutants showed an abnormal cardiac phenotype compared to 0% in Cas9 only controls (**p<0.01 vs controls). (**d**) Atrial end-systolic area was higher and ventricular end-diastolic area was lower in *nrap* and *fhod3ab* mutants compared to WT and Cas9 only controls (**p<0.01 versus controls). Data are shown as mean ± standard deviations of three independent experiments per sample, with each experiment including 3 technical replicates.

gnomAD, Genome Aggregation Database; WT, wild-type; mut, mutant; 2ΔΔCt, the relative fold

gene expression change between samples as a function of polymerase chain reaction thresholds.

Supplementary Figure 2

a

Gene Ontology

GO:MF Term name	Term ID	p_{adj}
actin binding	GO:0003779	2.822×10^{-10}
cytoskeletal protein binding	GO:0008092	1.171×10^{-8}
actin filament binding	GO:0051015	4.720×10^{-8}
structural constituent of muscle	GO:0008307	6.501×10^{-7}
troponin C binding	GO:0030172	5.342×10^{-5}
protein-containing complex binding	GO:0044877	5.813×10^{-4}
cell adhesive protein binding involved in bundle of His c...	GO:0086083	1.387×10^{-3}
cell-cell adhesion mediator activity	GO:0098632	4.184×10^{-3}
structural molecule activity	GO:0005198	6.091×10^{-3}
cell adhesion mediator activity	GO:0098631	6.969×10^{-3}
cell adhesion molecule binding	GO:0050839	7.039×10^{-3}
kinase binding	GO:0019900	7.220×10^{-3}
protein binding involved in heterotypic cell-cell adhesion	GO:0086080	7.613×10^{-3}
enzyme binding	GO:0019899	2.529×10^{-2}
calmodulin binding	GO:0005516	3.230×10^{-2}
protein kinase binding	GO:0019901	3.970×10^{-2}

KEGG

KEGG Term name	Term ID	p_{adj}
Hypertrophic cardiomyopathy	KEGG:05410	2.089×10^{-12}
Dilated cardiomyopathy	KEGG:05414	3.660×10^{-12}
Adrenergic signaling in cardiomyocytes	KEGG:04261	4.503×10^{-7}
Cardiac muscle contraction	KEGG:04260	3.704×10^{-6}
Arrhythmogenic right ventricular cardiomyopathy	KEGG:05412	1.815×10^{-5}

Reactome

REAC Term name	Term ID	p_{adj}
Muscle contraction	REAC:R-HSA-3...	2.927×10^{-9}
Striated Muscle Contraction	REAC:R-HSA-3...	4.016×10^{-8}
Ion homeostasis	REAC:R-HSA-5...	3.541×10^{-2}

b

Gene Ontology

GO:MF Term name	Term ID	p_{adj}
cell adhesive protein binding involved in bundle of His c...	GO:0086083	7.195×10^{-10}
protein binding involved in heterotypic cell-cell adhesion	GO:0086080	4.733×10^{-8}
cell-cell adhesion mediator activity	GO:0098632	1.867×10^{-7}
cell adhesion mediator activity	GO:0098631	4.464×10^{-7}
cell adhesion molecule binding	GO:0050839	7.916×10^{-5}
FATZ binding	GO:0051373	3.335×10^{-3}
alpha-catenin binding	GO:0045294	1.309×10^{-2}
actin binding	GO:0003779	3.563×10^{-2}
type III transforming growth factor beta receptor binding	GO:0034714	4.927×10^{-2}

KEGG

KEGG Term name	Term ID	p_{adj}
Arrhythmogenic right ventricular cardiomyopathy	KEGG:05412	2.522×10^{-5}
FoxO signaling pathway	KEGG:04068	5.001×10^{-4}
Renal cell carcinoma	KEGG:05211	2.929×10^{-3}
Chronic myeloid leukemia	KEGG:05220	4.091×10^{-3}
Hypertrophic cardiomyopathy	KEGG:05410	4.769×10^{-3}
Colorectal cancer	KEGG:05210	5.922×10^{-3}
Gastric cancer	KEGG:05226	1.366×10^{-2}
Acute myeloid leukemia	KEGG:05221	2.162×10^{-2}
Insulin signaling pathway	KEGG:04910	2.357×10^{-2}
Hepatitis B	KEGG:05161	3.855×10^{-2}
Hepatocellular carcinoma	KEGG:05225	4.140×10^{-2}

Reactome

REAC Term name	Term ID	p_{adj}
Formation of the cornified envelope	REAC:R-HSA-6...	1.285×10^{-2}
Apoptotic cleavage of cell adhesion proteins	REAC:R-HSA-3...	1.939×10^{-2}

Supplementary Figure 2: Pathways enriched for protein-coding and regulatory variants in the discovery cohort (n=209). (**a**) Gene Ontology (molecular function category), KEGG, and Reactome pathways enriched for pathogenic protein-coding and splicing variants. (**b**) Pathways enriched for high-risk regulatory variants including cell adhesion and binding categories.

Supplementary Figure 3

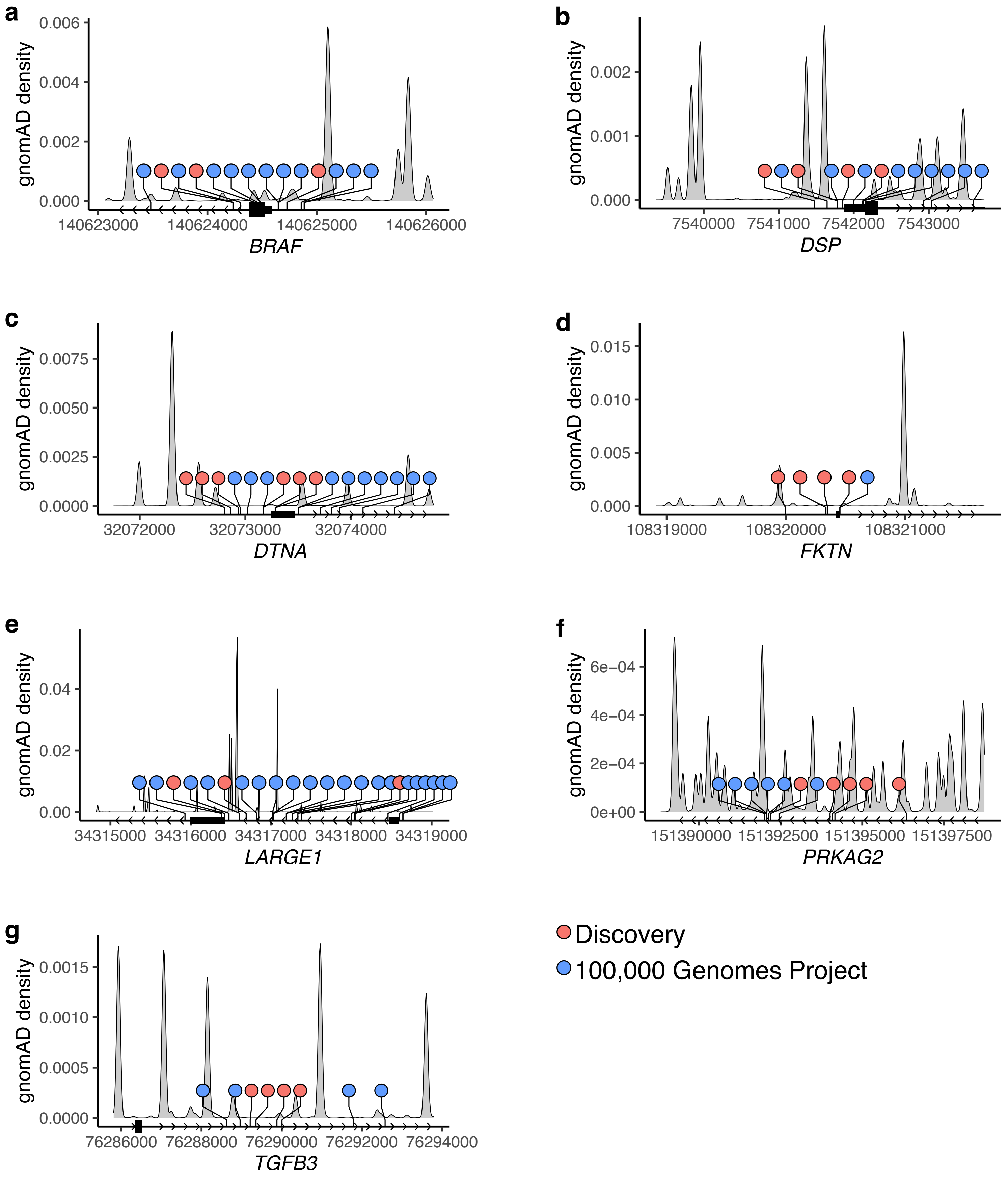

Supplementary Figure 3: Genomic location of variants in regulatory elements of genes prioritized for functional studies. The panels show the genomic coordinates of SNVs in the discovery cohort (n=209, orange dots) and the 100,000 Genomes Project replication cohort (n=1266, blue dots) mapped relative to the first (P1) promoter region and transcription start site for the following genes (**a**) *BRAF*, (**b**) *DSP*, (**c**) *DTNA*, (**d**) *FKTN*, (**e**) *LARGE1*, and to the enhancer regions for (**f**) *PRKAG2* (E15), and (**h**) *TGFB3* (E1). SNVs observed in gnomAD reference samples are plotted as grey density curves across the region. All regulatory variants were observed with an allele frequency <0.01% in gnomAD dataset, and tended to cluster in regions that were depleted for variants in gnomAD. Coordinates are based on hg19 reference genome.

gnomAD, Genome Aggregation Database.

Supplementary Figure 4

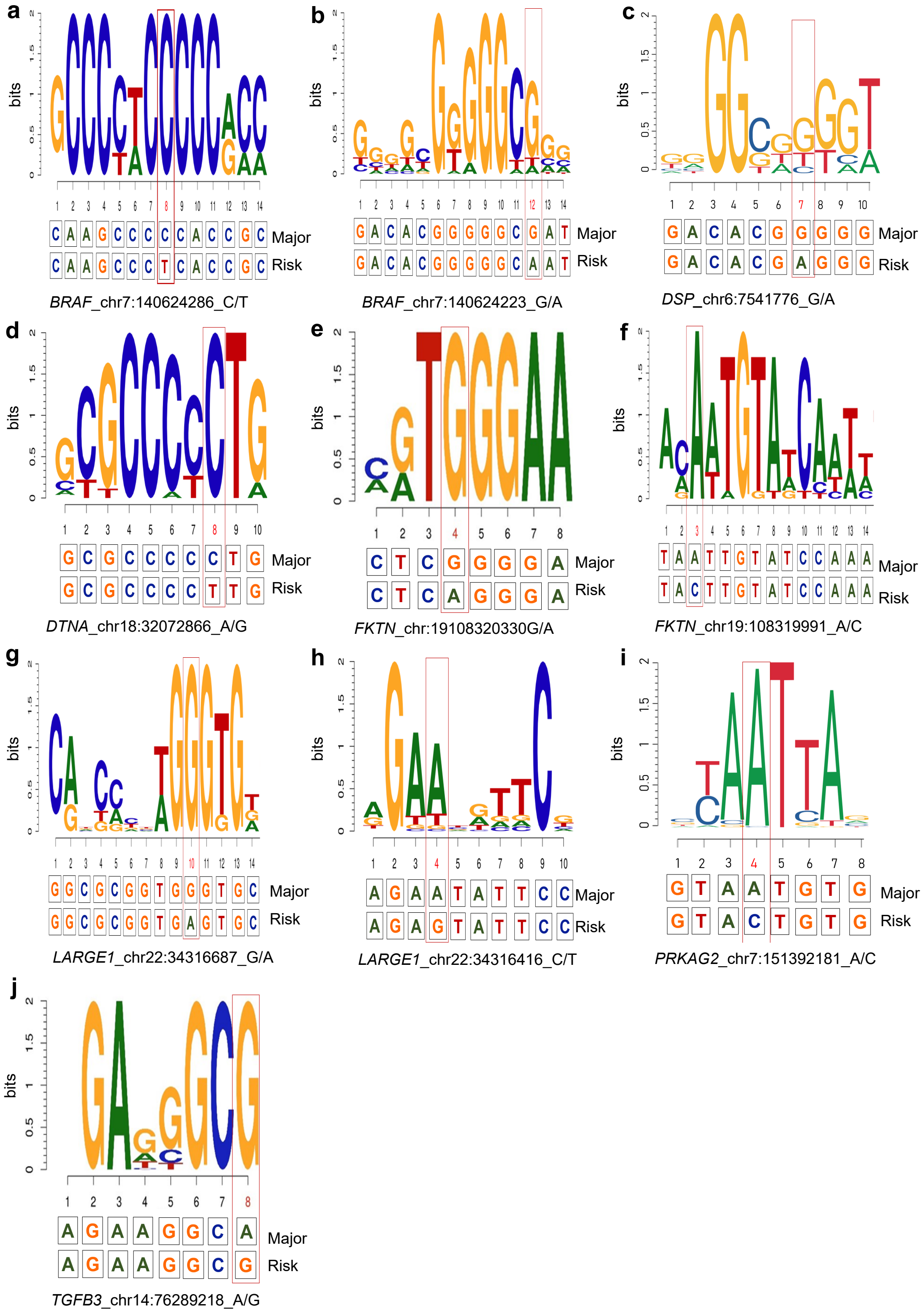

Supplementary Figure 4: Prediction effect of regulatory variants on transcription factor binding motif. SeqLogo was used to predict motif disruption caused by variants in the regulatory elements of (**a, b**) *BRAF*, (**c**) *DSP*, (**d**) *DTNA,* (**e**) *FKRP,* (**f, g**) *FKTN,* (**h, i**) *LARGE1,* (**j**) *PRKAG2,* and (**k**) *TGFB3*. The nucleotide base pair outlined in the red box indicates the position of the variant in the motif. Regulatory sequence analysis of variants shows a single nucleotide change in each variant compared to reference sequence resulting in a disruption in transcription factor motifs that is predicted to be associated with transcriptional up- or down-regulation of the target gene.

Major, reference sequence; Risk, variant sequence.

Supplementary Figure 5

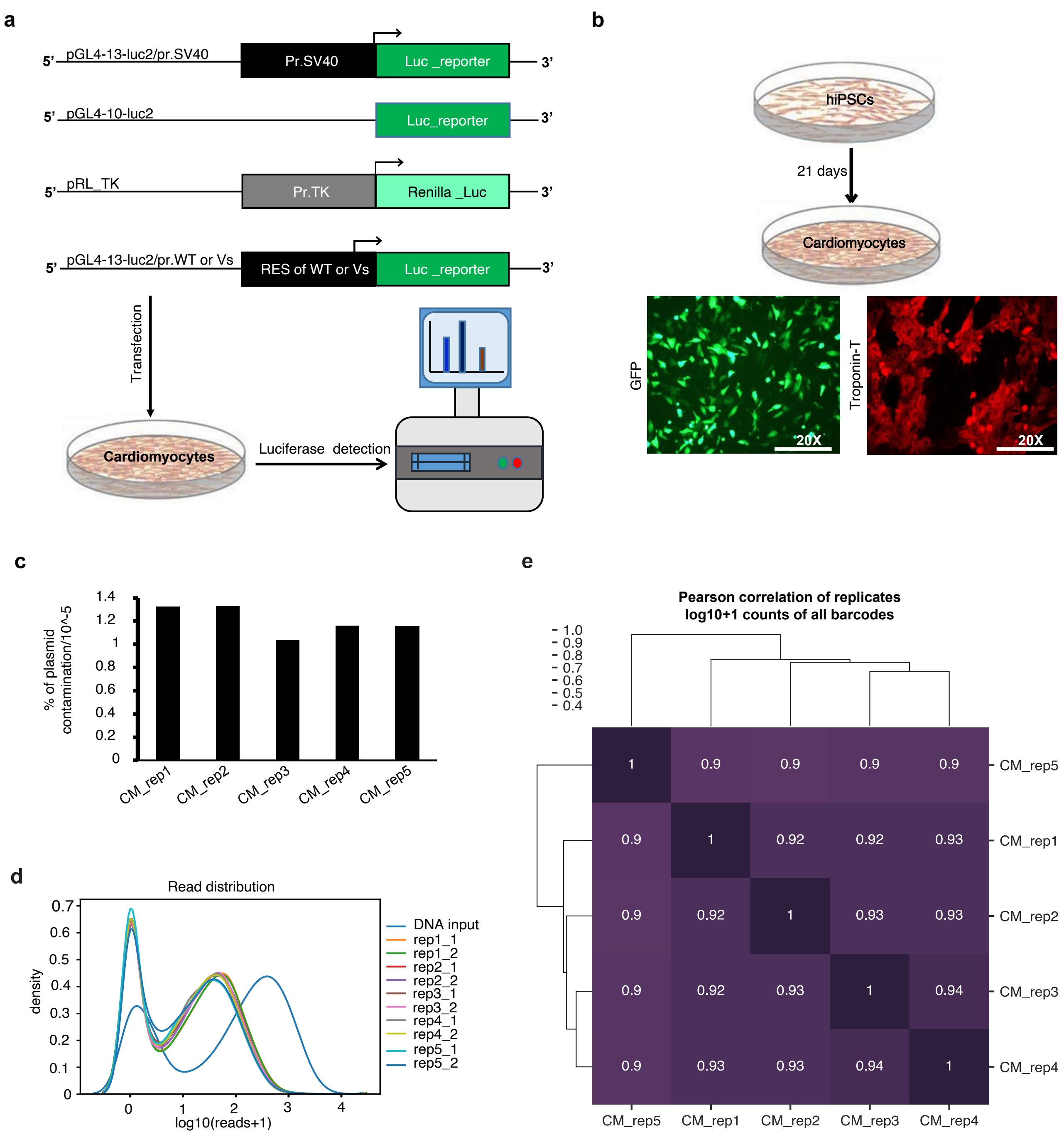

Supplementary Figure 5: Luciferase assays in hiPSC-derived CMs. (**a**) Luciferase reporter gene vectors harboring various promoter sequences were used. The promoter-driven control Firefly luciferase vector (pGL4-13_luc2_SV40) and Promoterless Firefly Luciferase Basic Vector (pGL4-10-luc2) were used as a positive and a negative control, respectively. Renilla Luciferase control reporter vectors (pRL_TK Vector) was used for normalization of transfection conditions. Sequence of regulatory elements of the predicted variants and wild-type were commercially synthesized and cloned separately into multiple cloning sites of Firefly Luciferase basic vectors, pGL4.10_luc2. hiPSC derived CMs were co-transfected with firefly luciferase vector harboring regulatory sequences separately and Renilla Luciferase control reporter vector. Luminescence was detected with Dual-Luciferase® Reporter (DLR™) assay system. (**b**) Successful differentiation (cardiac troponin T staining in red) and transfection of plasmid pX601_GFP (green) into day 21 PGP17 iPSC-derived cardiomyocytes using Lipofectamine Stem Transfection Reagent. Magnification: ×20. (**c**) qRT-PCR was performed to detect DNA contamination from plasmidpool transfection of 5 biological replicates of PGP17 cardiomyocytes. (**d**) Unimodal distribution of barcodes that represent the oligonucleotides used in this project. DNA input represents plasmid pool of oligonucleotides, whilst replicates 1-5 (each replicate split on two lanes of HiSeq2500) flowcell are tag-seq libraries derived from the transfections in cardiomyocytes. (**e**) Pearson correlation of 5 MPRA replicates.

hiPSC, human induced pluripotent stem cell; CM, cardiomyocytes; GFP, green fluorescent protein; P, plasmid; Pr, promoter; En, Enhancer; Luc, Luciferase; RES, Regulatory element sequence, WT, wild-type; V, Variant; rep, replicate.

Supplementary Figure 6

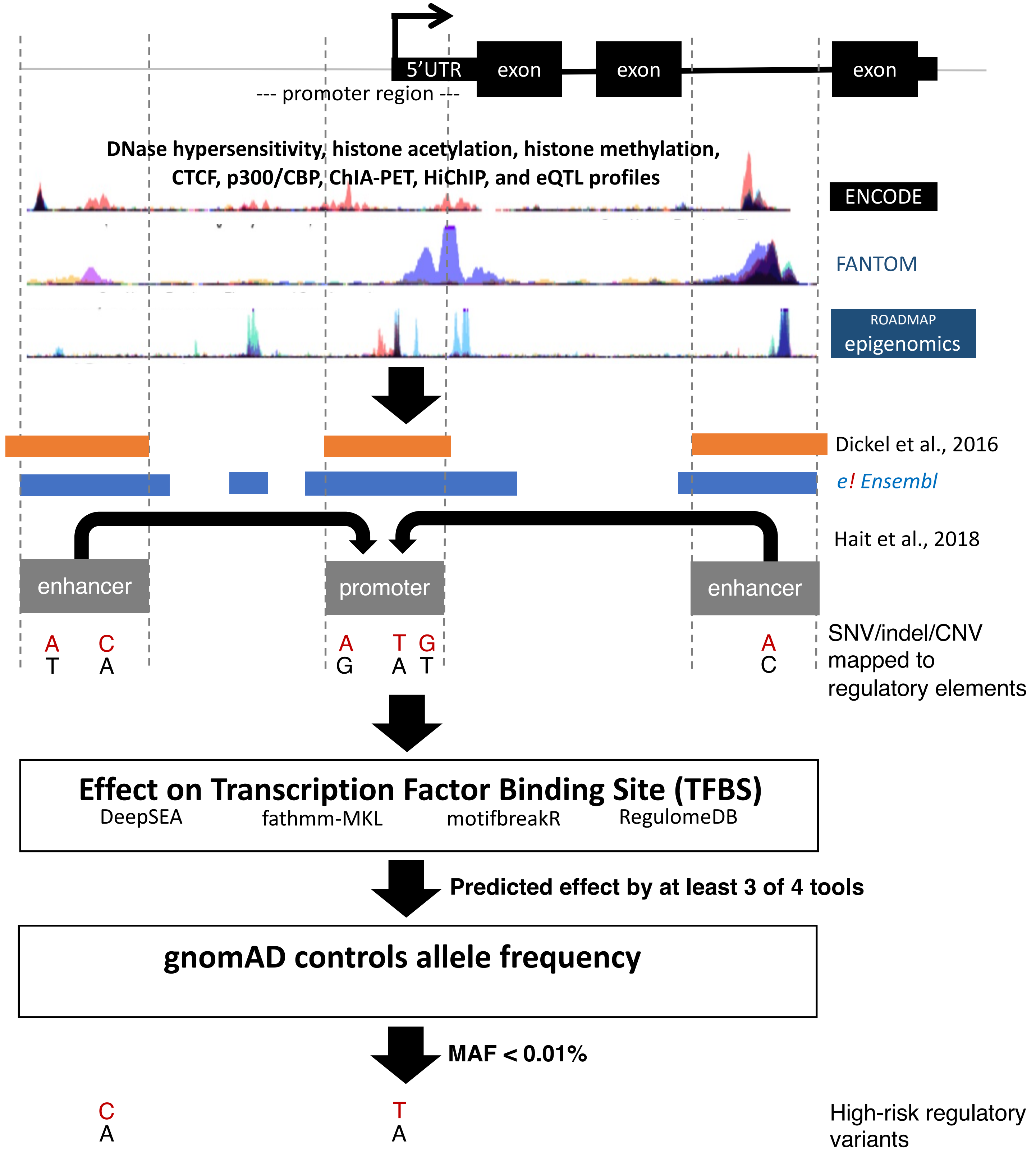

Supplementary Figure 6: Schematic representation of regulatory variant identification for a single gene: Exons are represented by black rectangles, with the transcription start site denoted by the right-facing arrow. Functionally active in human tissues were derived using experimental data from ENCODE, FANTOM, and Roadmap Epigenomics. These data were summarized and intersected in the Ensembl Regulatory Build and Dickel et al., 2016 to predict discrete regulatory regions. Promoters included regions 1.5kb upstream to 1kb downstream of gene transcription start sites, while enhancers were identified in intronic and nearby intergenic regions. Enhancers were mapped to gene promoters by genomic distance and using the database provided by Hait et al., 2018. Variants in these regulatory promoters and enhancers were further filtered based on predicted effect on transcription factor binding site activity by at least 3 of 4 computational tools, and minor allele frequency in the gnomAD control population (allele frequency < 0.01%).
UTR, untranslated region; SNV, single nucleotide variant; indel, insertion or deletion; CNV, copy number variant; gnomAD, Genome Aggregation Database; MAF, minor allele frequency.

SUPPLEMENTARY TABLES

Supplementary Table 1: Cardiomyopathy genes harboring pathogenic or likely pathogenic coding SNVs and indels (n=209 unrelated cases).

Supplementary Table 2: Copy number variants affecting cardiomyopathy genes (n=209 unrelated cases).

Supplementary Table 3: Loss of function variants in other candidate cardiomyopathy genes in discovery (n=209) and replication (n=1266) cohorts.

Supplementary Table 4: High-risk (and candidate) regulatory SNVs in regulatory elements of cardiomyopathy genes (n=209 cases).

Supplementary Table 5: Massively parallel reporter assay for regulatory SNVs in regulatory elements of cardiomyopathy genes.

Supplementary Table 6: Clinical characteristics of pediatric cardiomyopathy probands in the discovery cohort (n=209).

Supplementary Table 7. Cardiomyopathy genes (n=84) with annotations.

Supplementary Table 8. Regulatory regions associated with 84 CMP genes (plus *LARGE1*).

Supplementary Table 9: Normalized RNAseq data for genes with high-risk CNVs, LoF and regulatory variants.

Supplementary Table 10: Primer pairs for myocardial and zebrafish studies.

Supplementary Table 11: Antibodies for Western blot and immunohistochemistry.

Supplementary Table 12: Synthesis of gene promoter and enhancer sequences for luciferase assays.

Supplementary Table 13: Design of single guide RNAs to target nrap and fhod3ab in zebrafish embryos.
