# Supplementary material for: Whole genome sequencing delineates regulatory, copy number, and cryptic splice variants in early onset cardiomyopathy
Source: NPJ Genom Med. 2022 Mar 14;7:18. doi: 10.1038/s41525-022-00288-y (PMC8921194; doi:10.1038/s41525-022-00288-y)
Supplement: Supplementary file 3 — Reporting Summary [file 41525_2022_288_MOESM3_ESM.pdf]

nature research

Corresponding author(s): Seema Mital

Last updated by author(s): Jan 14, 2022

Reporting Summary

Nature Research wishes to improve the reproducibility of the work that we publish. This form provides structure for consistency and transparency in reporting. For further information on Nature Research policies, see our Editorial Policies and the Editorial Policy Checklist.

Statistics

For all statistical analyses, confirm that the following items are present in the figure legend, table legend, main text, or Methods section.

n/a	Confirmed	
☐	☒	The exact sample size (*n*) for each experimental group/condition, given as a discrete number and unit of measurement
☐	☒	A statement on whether measurements were taken from distinct samples or whether the same sample was measured repeatedly
☐	☒	The statistical test(s) used AND whether they are one- or two-sided<br>*Only common tests should be described solely by name; describe more complex techniques in the Methods section.*
☒	☐	A description of all covariates tested
☐	☒	A description of any assumptions or corrections, such as tests of normality and adjustment for multiple comparisons
☐	☒	A full description of the statistical parameters including central tendency (e.g. means) or other basic estimates (e.g. regression coefficient) AND variation (e.g. standard deviation) or associated estimates of uncertainty (e.g. confidence intervals)
☐	☒	For null hypothesis testing, the test statistic (e.g. *F*, *t*, *r*) with confidence intervals, effect sizes, degrees of freedom and *P* value noted<br>*Give P values as exact values whenever suitable.*
☒	☐	For Bayesian analysis, information on the choice of priors and Markov chain Monte Carlo settings
☐	☒	For hierarchical and complex designs, identification of the appropriate level for tests and full reporting of outcomes
☐	☒	Estimates of effect sizes (e.g. Cohen's *d*, Pearson's *r*), indicating how they were calculated

Our web collection on statistics for biologists contains articles on many of the points above.

Software and code

Policy information about availability of computer code

Data collection	Whole genome sequencing was performed on Illumina HiSeq X platform, and RNAseq was performed using the Illumina HiSeq 2500 platform.
Data analysis	Discovery cohort whole genome sequencing alignments were made with Isaac aligner, followed by short variant calling using the Isaac variant caller. WGS quality metrics were calculated using mosdepth (https://github.com/brentp/mosdepth). Variants were annotated using snpEff, annovar, and VEP. Short variants were filtered using bcftools and lifted over between reference versions using Picard LiftoverVcf. de novo variants were identified using the Genome Analysis Toolkit (GATK) (v4.1.2.0). CNV calls were made using ERDS (v1.1) and CNVnator (v0.3.2). RNAseq samples were aligned using Tophat (v.2.0.11) and processed to extract raw read counts for genes using htseq-count (v.0.6.1p2). Sequencing data were mapped to the human transcriptome using HISAT2 spliced aligner, and gene expression level was quantified using StringTie. Pathogenicity of missense variants was predicted using SIFT, PolyPhen2, MutationTaster2, Mutation Assessor, CADD, PROVEAN, PHAST, MetaSVM, and MetaLR. Genomic conservation scores were obtained from GERP++ and phastCons. Putative protein-truncating variants predicted to cause loss of function including splice-site, nonsense and frameshift variants were assessed and annotated using LOFTEE tool (https://github.com/konradjk/loftee) as a plugin via Ensembl's Variant Effect Predictor (VEP v90) tool. InterVar was used to confirm pathogenicity calls. Transcription factor binding site perturbations were identified using RegulomeDB, motifbreakR, DeepSEA, and Fathmm-MKL. Pathway enrichment analysis was performed using g:Profiler (https://biit.cs.ut.ee/gprofiler). All statistical analyses were done using R statistical software (v3.5.1). All computational tools used in this study are available as commercial or open-source software.

For manuscripts utilizing custom algorithms or software that are central to the research but not yet described in published literature, software must be made available to editors and reviewers. We strongly encourage code deposition in a community repository (e.g. GitHub). See the Nature Research guidelines for submitting code & software for further information.

Data

Policy information about availability of data

All manuscripts must include a data availability statement. This statement should provide the following information, where applicable:

- Accession codes, unique identifiers, or web links for publicly available datasets
- A list of figures that have associated raw data
- A description of any restrictions on data availability

Sequencing data are deposited in the EGA European Genome-Phenome Archive and are available under accession EGAS00001004929 upon approval by the Data Access Committee.

Field-specific reporting

Please select the one below that is the best fit for your research. If you are not sure, read the appropriate sections before making your selection.

☒ Life sciences ☐ Behavioural & social sciences ☐ Ecological, evolutionary & environmental sciences

For a reference copy of the document with all sections, see nature.com/documents/nr-reporting-summary-flat.pdf

Life sciences study design

All studies must disclose on these points even when the disclosure is negative.

Sample size	The sample size was a convenience sample size based on the availability of eligible patients in our biobank accrued across a time span of 10 years from 7 centers across the province of Ontario. Our cohort represents one of the largest biobanked cohorts of childhood cardiomyopathy, a rare disorder. For controls, we accessed 1326 cancer individuals without heart disease from the ICGC consortium who had sample level sequencing data available through an institutional agreement. The statistical methods for case-control burden analyses are described in the manuscript.
Data exclusions	Patients with secondary cardiomyopathy resulting from inborn errors of metabolism, mitochondrial disorders, syndromic, and neuromuscular etiologies were excluded.
Replication	The identification of regulatory SNVs and LoF variants in novel genes were replicated in an independent set of 1266 CMP cases obtained from the 100,000 Genomes Project. Replication cases were selected based on being probands with CMP as a primary diagnosis, and not having any additional syndromic phenotypes noted. LoF variants were additionally replicated in independent cohorts of 587 Australian CMP cases and 100 South Asian CMP cases.
Randomization	Not applicable.
Blinding	All data was de-identified. Investigators were aware of sample diagnosis, which was required to perform analyses.

Reporting for specific materials, systems and methods

We require information from authors about some types of materials, experimental systems and methods used in many studies. Here, indicate whether each material, system or method listed is relevant to your study. If you are not sure if a list item applies to your research, read the appropriate section before selecting a response.

Materials & experimental systems

n/a	Involved in the study	
☐	☒	Antibodies
☐	☒	Eukaryotic cell lines
☒	☐	Palaeontology and archaeology
☐	☒	Animals and other organisms
☐	☒	Human research participants
☒	☐	Clinical data
☒	☐	Dual use research of concern

Methods

n/a	Involved in the study	
☒	☐	ChIP-seq
☒	☐	Flow cytometry
☒	☐	MRI-based neuroimaging

Antibodies

Antibodies used	Details available in Supplementary table 12.<br>1- FKTN: Rabbit monoclonal [EPR7913] to Fukutin, Abcam, Ab131280<br>2- LARGE1: Anti-LARGE Polyclonal, Thermo Fisher Scientific, PA5-78393<br>3- PRKAG2: Rabbit PRKAG2 Polyclonal Antibody, Mybiosource, MBS9134285<br>4- TGFB3: Anti-TGF beta 3 antibody, Abcam, Ab15537

	5- NRAP: Anti-NRAP Polyclonal, Thermo Fisher Scientific, PA5-88772<br>6- GAPDH: Anti-GAPDH antibody [6C5], Abcam, Ab8245<br>7- Tropnin T: Anti-Cardiac Troponin T antibody [1F11], Abcam, ab10214<br>8- Goat Anti-Mouse IgG H&L (Alexa Fluor® 647), Abcam, ab150115<br>9- Mouse IgG HRP-conjugated Antibody, R&D Systems, HAF007<br>10- Rabbit IgG HRP-conjugated Antibody, R&D Systems, HAF008
Validation	Details available in Supplementary table 12.<br>1- FKTN: https://www.abcam.com/fukutin-antibody-epr7913-ab131280.html<br>2- LARGE1: https://www.thermofisher.com/antibody/product/LARGE-Antibody-Polyclonal/PA5-78393<br>3- PRKAG2: https://www.mybiosource.com/polyclonal-human-mouse-rat-antibody/prkag2/9134285<br>4- TGFB3: https://www.abcam.com/tgf-beta-3-antibody-ab15537.html<br>5- NRAP: https://www.thermofisher.com/antibody/product/NRAP-Antibody-Polyclonal/PA5-88772<br>6- GAPDH: https://www.abcam.com/gapdh-antibody-6c5-loading-control-ab8245.html<br>7- Troponin T: https://www.abcam.com/cardiac-troponin-t-antibody-1f11-ab10214.html<br>8- Alexa Fluor® 647: https://www.abcam.com/goat-mouse-igg-hl-alexa-fluor-647-ab150115.html<br>9- Mouse IgG HRP: https://www.rndsystems.com/products/mouse-igg-hrp-conjugated-antibody_haf007<br>10- Rabbit IgG HRP: https://www.rndsystems.com/products/rabbit-igg-hrp-conjugated-antibody_haf008

Eukaryotic cell lines

Policy information about cell lines

Cell line source(s)	PGP17 induced pluripotent stem cells generated from healthy individuals enrolled in the Personal Genome Project Canada were used for our study. The donors have consented to sharing of their cells and will be available on reasonable request (Reuter MS et al, CMAJ 2018 - PMID 29431110; Hildebrandt MR et al, Stem Cell Reports 2019 - PMID: 31813827)
Authentication	The PGPC17 donor and the derived iPSCs were both whole genome sequenced confirming their identity (Hildebrandt et al, Stem Cell Reports 2019 - PMID: 31813827)
Mycoplasma contamination	PGPC17 iPSCs used in our study tested negative for mycoplasma contamination.
Commonly misidentified lines<br>(See ICLAC register)	No misidentified cell lines were used in our study.

Animals and other organisms

Policy information about studies involving animals; ARRIVE guidelines recommended for reporting animal research

Laboratory animals	Following standard procedures, pair matings of adult transgenic Tg(myl7::GFP) (~3 months) male and female zebrafish were set up to recover one-cell stage embryos for co-injections of gRNAs and CRISPR-Cas9 (www.zfin.org/zf_info/zfbook/chapt2/2.1.html<http://www.zfin.org/zf_info/zfbook/chapt2/2.1.html<http://www.zfin.org/zf_info/zfbook/chapt2/2.1.html<http://www.zfin.org/zf_info/zfbook/chapt2/2.1.html>>). For cardiac imaging, 3 day post-fertilization embryos were used.
Wild animals	The study did not involve wild animals.
Field-collected samples	The study did not involve samples collected from the field.
Ethics oversight	Zebrafish protocols used in this study were approved by the SickKids Animal Care Committee (Protocol #401951).

Note that full information on the approval of the study protocol must also be provided in the manuscript.

Human research participants

Policy information about studies involving human research participants

Population characteristics	The primary or discovery cohort comprised 209 unrelated primary CMP index cases less than 21 years old at diagnosis, and affected and unaffected family members. HCM, DCM, RCM, LVNC and AVC were diagnosed based on published clinical criteria. Patients with secondary CMPs resulting from inborn errors of metabolism, mitochondrial disorders, syndromic, and neuromuscular etiologies were excluded. Clinical data including demographics, diagnosis, family history, clinical genetic testing results, and outcomes during follow-up were captured. The median age at phenotypic onset was 5.2 years, 57.9% were male. Major ethnicities were 67% White, 19% Asian, 10% Black. 31% of cases were genotype-positive on previous clinical panel testing, 16% were clinically untested. Ten cases (4.8%) died, and 126 cases (60.3%) experienced a major adverse cardiac event on follow-up. Details are provided in the Supplementary Table 6.
Recruitment	Eligible participants in the discovery cohort were prospectively enrolled in the Heart Centre Biobank Registry at The Hospital for Sick Children, Toronto (https://theheartcentrebiobank.com/) with consent to collect clinical data and biospecimens including DNA, myocardial samples, and skin or blood samples for reprogramming.
Ethics oversight	Hospital for Sick Children, Children's Hospital of Eastern Ontario, Toronto General Hospital, London Health Sciences Centre, Kingston General Hospital, and Hamilton Health Sciences Centre

Note that full information on the approval of the study protocol must also be provided in the manuscript.
